# A DNA dual lock-and-key strategy for cell-subtype-specific siRNA delivery

**DOI:** 10.1038/ncomms13580

**Published:** 2016-11-24

**Authors:** Kewei Ren, Ying Liu, Jie Wu, Yue Zhang, Jing Zhu, Min Yang, Huangxian Ju

**Affiliations:** 1 State Key Laboratory of Analytical Chemistry for Life Science, School of Chemistry and Chemical Engineering, Nanjing University, Nanjing 210023, China; 2 Department of Pharmaceutical and Biological Chemistry, UCL School of Pharmacy, University College London, London WC1N 1AX, UK

## Abstract

The efficient and precise delivery of siRNA to target cells is critical to successful gene therapy. While novel nanomaterials enhance delivery efficiency, it still remains challenging for precise gene delivery to overcome nonspecific adsorption and off-target effect. Here we design a dual lock-and-key system to perform cell-subtype-specific recognition and siRNA delivery. The siRNA is self-assembled in an oligonucleotide nano vehicle that is modified with a hairpin structure to act as both the ‘smart key’ and the delivery carrier. The auto-cleavable hairpin structure can be activated on site at target cell membrane by reacting with two aptamers as ‘dual locks’ sequentially, which leads to cell-subtype discrimination and precise siRNA delivery for high efficient gene silencing. The success of this strategy demonstrates the precise delivery of siRNA to specific target cells by controlling multiple parameters, thus paving the way for application of RNAi in accurate diagnosis and intervention.

## Abstract (web summary)

Delivery of siRNA to target cells is essential for *in vivo* gene therapy. Here the authors demonstrate an oligonucleotide aptamer that targets therapeutic siRNA to a specific cell type.

## Graphical abstract

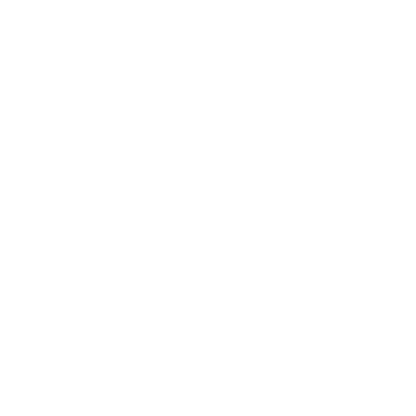

Gene interference technology that selectively silences gene expression and inhibits protein transcription by delivering small interference RNA (siRNA) in mammalian cells is becoming a promising approach for the precise treatment of human diseases, including cancer and metabolic, neurodegenerative and infectious diseases^1^,^2^. One of the key challenges to realize the broad clinical application of RNA interference (RNAi) therapy is its delivery specificity^3^,^4^,^5^. A cell-specific and efficient delivery system is highly desired to improve selective cellular uptake, decrease the overall dosage of siRNAs and avoid nonspecific adsorption as well as minimize off-target silencing in non-target cells^6^. A number of ligands that selectively bind tissue-associated antigens have been explored for targeted siRNA delivery, including antibody–protamine fusion proteins^7^,^8^ and aptamer-siRNA chimeras^9^,^10^. However, most receptors are often shared by multiple types of cell, or a receptor overexpressed in diseased cells is also expressed at a low level in normal cells, therefore the single-receptor-targeted delivery system potentially results in off-target toxicities and serious complications^11^. Since cells express multiple surface receptors, simultaneously assessing multiple surface receptors to recognize specific disease cells and enhance diagnostic and therapic accuracy in similar cells should be a more practical and less risk approach^12^. Taking advantages of autonomous DNA strand displacement cascades reaction^13^, programmable dual parameters controlled DNA logic platform has been used for cancer cell recognition^14^ and photodynamic therapy^15^,^16^. However, the DNA logic platform has not been used for siRNA delivery due to the limitation of using toehold-mediated strand displacement cascade reaction as an efficient delivery carrier. The precise delivery of siRNA to specific target cells is still an urgent need.

A variety of materials have been explored as siRNA delivery carriers, such as liposomes, cationic polyelectrolytes and inorganic nanoparticle^17^,^18^,^19^. However, these conventional delivery vehicles suffer from low loading efficiency, less cell-specific manner, complex surface modification process and/or the damage of immunogenic response or toxicity^20^,^21^. Self-assembled DNA nanostructures can offer the advantages of flexible design, controllable size and orientation, ease of bioconjugation and excellent biocompatibility, and have demonstrated potential application in biosensing and drug delivery^22^,^23^,^24^. Here a self-assembled oligonucleotide nano vehicle (ONV) and a ‘dual lock-and-key’’ were designed to load siRNA for controllable siRNA delivery. The ONV structure conferred higher payload capacity, which significantly increased cell uptake. Besides, different cell-recognition aptamers could be conveniently incorporated into ONV via hybridization, and the rigid tube-like structure improved resistance to nuclease degradation upon endocytosis^25^,^26^.

The incorporation of two factors in a delivery system to function in a serial manner can improve the site-specific transport and lower the non-target cytotoxicity^27^. Here an auto-cleavable hairpin structure is used to modify the siRNA-loaded ONV (siRNA-ONV) and act as the ‘smart key’, and two kinds of aptamers, sgc8c and sgc4f^16^, are bound on cell surface to act as the ‘double locks’. The ‘locks’ can be opened sequentially by reacting with the ‘key’ in a serial manner. The specific double recognition mode controls the cell ‘locked-open’ status, and thus achieves cell-subtype-specific recognition and precise siRNA delivery. Upon reaction with Zn^2+^-dependent MNAzyme on sgc4f, the hairpin structure oligonucleotide in siRNA-ONV is auto-cleaved to form single strand, which activates the ‘smart key’ on site at cell membrane. The ‘locked’ hairpin structure of sgc8c is thus opened by hybridizing with the cleaved single-strand oligonucleotide in siRNA-ONV subsequently to mediate the precise delivery of siRNA into specific target cells. To the best of our knowledge, this is the first try of precise siRNA delivery and gene silencing with the participation of multiple cell membrane receptors. Previously reported siRNA delivery methods all rely on only one receptor on cell surface^7^,^8^,^9^,^10^, therefore usually suffer from the high nonspecific interaction and off-target toxicity to other cells. In comparison with ‘single-parameter’-controlled siRNA delivery, the ‘dual lock-and-key’-controlled siRNA delivery system activates two recognition elements on site at target cell membrane just before the delivery process, therefore affords substantial improvement for delivery specificity and avoids off-target toxicities, which is of great importance to the application of RNAi in precise diagnosis and treatment.

## Results

### Preparation siRNA-ONV

**Figure 1:**
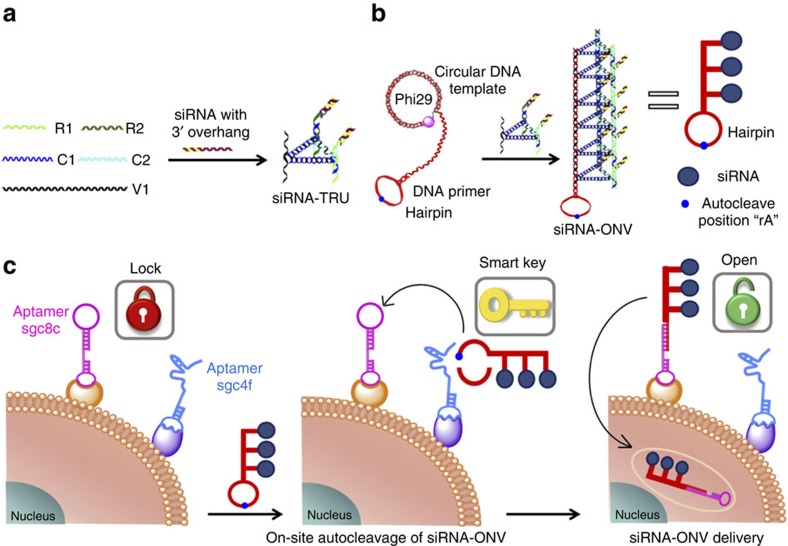
Schematic illustration of ‘dual lock-and-key’-controlled cell-subtype-specific siRNA delivery. (**a**) Self-assembly synthesis of siRNA-TRU repeating unit, (**b**) self-assembly synthesis of siRNA-ONV nanotube and (**c**) siRNA delivery principle.

**Figure 2:**
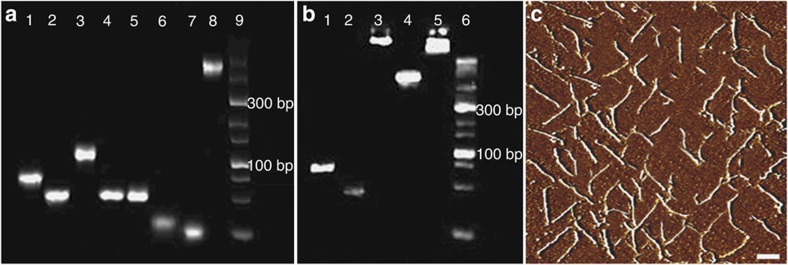
Characterization of siRNA-ONV self-assembly. (**a**) PAGE analysis of siRNA-TRU self-assembly. Lanes 1–9 represent C1, C2, V1, R1, R2, antisense siRNA, siRNA, the mixture of lines 1–7 and DNA ladder marker. (**b**) PAGE analysis of siRNA-ONV self-assembly. Lanes 1–6 represent DNA primer, circular DNA template, DNA backbone, siRNA-TRU, siRNA-ONV and DNA ladder marker. (**c**) AFM image of siRNA-ONV. Phase image: scale bar, 300 nm.

The triangular rung units (TRUs) with two overhangs at each end were synthesized first as building blocks for ONV (Fig. 1a)^28^. siRNAs were hybridized with the overhangs of TRU, and the synthesis of siRNA-TRU was confirmed by 8% polyacrylamide gel electrophoresis (PAGE) experiment (Fig. 2a). Upon mixing seven oligonucleotides of C1, C2, V1, R1, R2, antisense siRNA and siRNA strands, a distinct bright band that migrated much slower than all components was observed (lane 8, Fig. 2a), indicating the successful synthesis of siRNA-TRU. The siRNA-TRUs were subsequently assembled with a long continuous DNA backbone strand produced by rolling circle amplification (RCA) to form siRNA-ONV (Fig. 1b). The synthesis process of siRNA-ONV was characterized in Fig. 2b, it showed a lower-mobility band appeared in lane 3 after the RCA reaction, suggesting the formation of DNA backbone. When DNA backbone was mixed with the siRNA-TRU (lane 4, Fig. 2b), the band for siRNA-TRU disappeared and a strengthened band was obtained in the sample loading zone, showing the successful preparation of siRNA-ONV (Fig. 2b, lane 5). The siRNA-ONV structure was also characterized by atomic force microscopy (AFM). The siRNA-ONV tubes appeared to be quite rigid and well dispersed with an average length of 0.60±0.15 μm (Fig. 2c) (± indicates the length range of siRNA-ONV tubes according to 10 times replicate measurements), which consists with the previously reported self-assembly DNA nanotube structure^28^,^29^. Given that the siRNA-TRU is 14.3 nm in length^30^, 42±10 siRNA-TRU repeating units were counted for each siRNA-ONV, indicating the loading capacity of 84±20 siRNA for siRNA-ONV.

To verify the serum stability of siRNA-ONV, Cy3-tagged siRNA and black hole quencher 2 (BHQ2)-tagged R1 and R2 oligonucleotides were used as components to assemble self-quenched siRNA-ONV (SQ-siRNA-ONV). The fluorescence recovery of Cy3-siRNA from SQ-siRNA-ONV disassembly was measured in 10% fetal bovine serum (FBS) reaction buffer over 12 h (Supplementary Fig. 1a). Compared with the control couple of self-quenched double-strand siRNA (SQ-ds-siRNA), the fluorescence recovery from SQ-siRNA-ONV was much less, demonstrating that ONV nanotube structure could protect siRNA from nuclease degradation. The melting temperature (*T*_m_) of siRNA-ONV was 72.1 °C (Supplementary Fig. 1b,c), demonstrating good thermal stability.

### Mechanism of double locks controlled siRNA delivery

The feasibility of dual parameter sequentially controlled ‘lock-and-key’ system was verified with PAGE analysis by mixing the hairpin structure oligonucleotide (DNA primer) in ONV with the ‘locks’ (sgc8c and sgc4f aptamers) together and respectively (Supplementary Fig. 2a). Although sgc8c aptamer and DNA primer had the complementary sequences, their individual ‘closed’ hairpin structure prevented the hybridization, demonstrated by two separate bands for the mixture solution (Supplementary Fig. 2a, lanes 4). The hairpin structure DNA primer had an auto-cleavable position, which could be autocatalytically cleaved by the Zn^2+^-dependent MNAzyme containing sgc4f aptamer to form DNA single strand. The cleaved single-strand DNA primer hybridized with sgc8c aptamer to open its hairpin structure, acting as a smart key to be activated on site by reacting sequentially with double locks sgc4f and sgc8c. Upon the addition of sgc4f aptamer in the mixture of sgc8c and DNA primer, the bands representing sgc8c and DNA primer disappeared, and a new band with much lower mobility appeared (Supplementary Fig. 2a, lanes 5), indicating the hybridization of single-strand DNA primer with sgc8c. The feasibility of the ‘dual lock-and-key’-controlled system was further confirmed by observing fluorescence recovery from self-quenched aptamer BHQ2-sgc8c. The hairpin structure aptamer sgc8c was labelled with Cy3 and BHQ2 on each stem terminus. The ‘locked’ sgc8c aptamer kept hairpin structure in the absence of sgc4f aptamer or/and ‘smart key’ ONV, thus only very weak fluorescence was observed. In the presence of both sgc4f and ONV, the mixture solution demonstrated strong fluorescent intensity due to the opening of hairpin structure sgc8c aptamer and corresponding Cy3 fluorescence recovery (Supplementary Fig. 2b).

Sgc8c and sgc4f aptamers are bound to their individual receptors expressed by different cells. Sgc8c binds to cell membrane receptor tyrosine protein kinase-7, while the cell membrane receptor for sgc4f is not yet identified^16^. After different cells were incubated with fluorescein dye (FAM) labelled aptamers, strong FAM fluorescence were observed from their corresponding target cells in flow cytometric assay (Supplementary Fig. 3), which indicated human cervix carcinoma (HeLa) cells only bound sgc8c aptamer, and human Burkitt’s lymphoma (Ramos) cells only bound sgc4f aptamer, while both sgc8c and sgc4f aptamers were bound to human acute lymphoblastic leukaemia (CEM) cells, indicating the binding selectivity of sgc8c and sgc4f to their respective target cells.

The ‘double locks’ controlled CEM cell-subtype-specific siRNA-ONV delivery was relied on the sequential opening of locks sgc8c and sgc4f (Fig. 1c). The fluorescence from Cy3-labelled siRNA-ONV (Cy3-siRNA-ONV) was only observed within CEM cells in the presence of both sgc8c and sgc4f aptamers, while no fluorescence was observed in the absence of either aptamer (Supplementary Fig. 4a). This result was also confirmed by flow cytometric assay (Supplementary Fig. 4b). Z-stack images of CEM cells were used to verify the internalization of Cy3-siRNA-ONV in CEM cells^31^. The fluorescence signals from Cy3 and the nucleus staining were located at the same focal plane and exhibited a position-sensitive dependence, demonstrating intracellular localization of Cy3-siRNA-ONV (Supplementary Fig. 4c).

### Specificity of dual lock-and-key-controlled siRNA delivery

**Figure 3:**
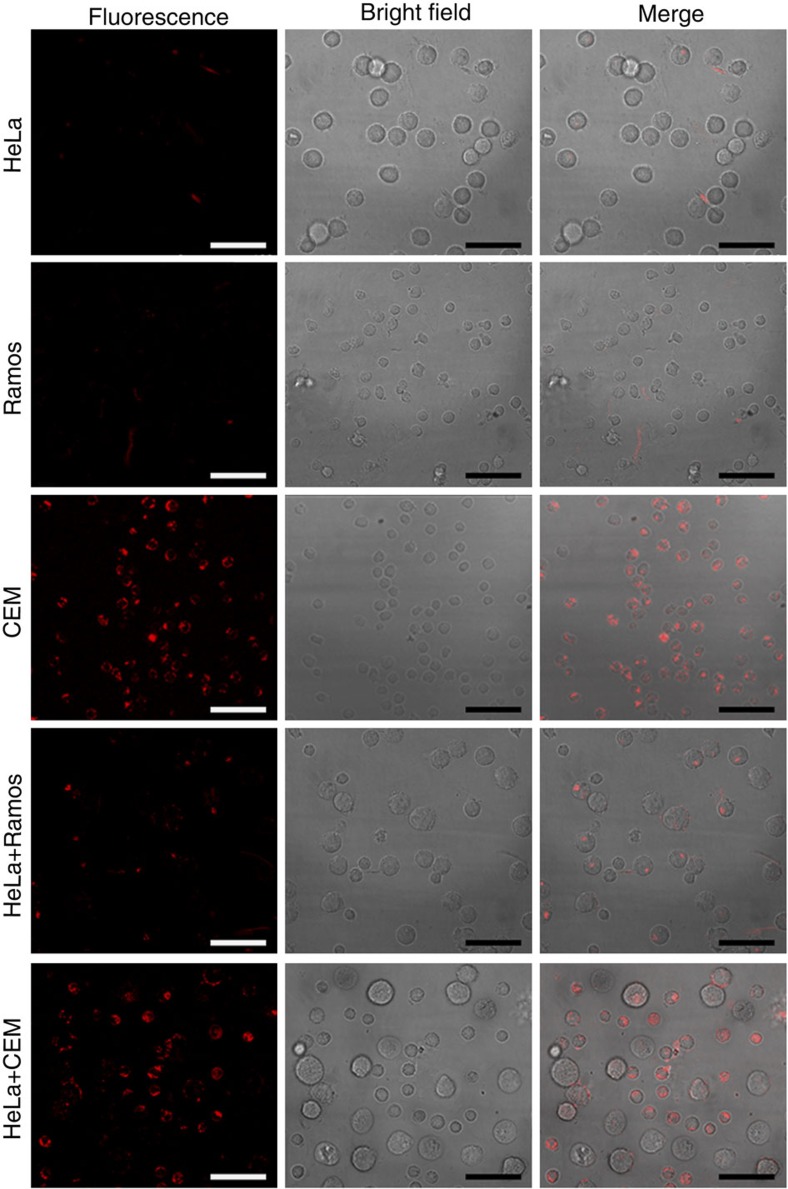
Specificity of ‘dual lock-and-key’-controlled siRNA delivery. Confocal microscopy images of HeLa, Ramos, CEM cells, the mixture of HeLa and Ramos cells, and the mixture of HeLa and CEM cells after incubation with 100 nM Cy3-siRNA-ONV for 2 h. All the cells were pretreated with sgc8c and sgc4f aptamers. Scale bars, 50 μm.

The cell-subtype recognition and siRNA-ONV delivery were further verified with three different cells, HeLa, Ramos and CEM cells (Fig. 3). The bright fluorescence from Cy3-siRNA-ONV was only observed within CEM cells. Only little fluorescence was observed from Ramos and HeLa cells, demonstrating the specific recognition and precise delivery of siRNA to target cells. To examine the possible off-target delivery through the diffusion of activated siRNA-ONV or direct physical contact of neighbouring cells, the mixture of HeLa and Ramos cells or HeLa and CEM cells was treated with Cy3-siRNA-ONV, respectively. Both HeLa and Ramos cells showed little fluorescence, while CEM cells showed bright fluorescence, indicating that proximity-based siRNA delivery was dominant. The fluorescence intensity of CEM cells was 4.6-fold that of HeLa cells (Supplementary Fig. 5), suggesting that ∼82.1% of activated siRNA-ONV in the mixture was internalized in CEM cells. To further demonstrate the advantages of dual parameters controlled cell-subtype-specific recognition and delivery, we also designed a single-parameter-controlled delivery vehicle as a negative control Cy3-siRNA-ONV (S-Cy3-siRNA-ONV). The ONV nanotube was modified with a single-strand DNA oligonucleotide instead of auto-cleavable hairpin structure oligonucleotide. The single-strand DNA oligonucleotide had the same sequence as the rest part of the auto-cleavable hairpin structure oligonucleotide after cleavage and could recognize lock aptamer sgc8c. However, the single-membrane receptor-controlled delivery system could not provide sufficient cell-subtype selectivity, fluorescence from S-Cy3-siRNA-ONV was observed from both CEM and HeLa cells (Supplementary Fig. 6).

### Internalization process of siRNA-ONV

**Figure 4:**
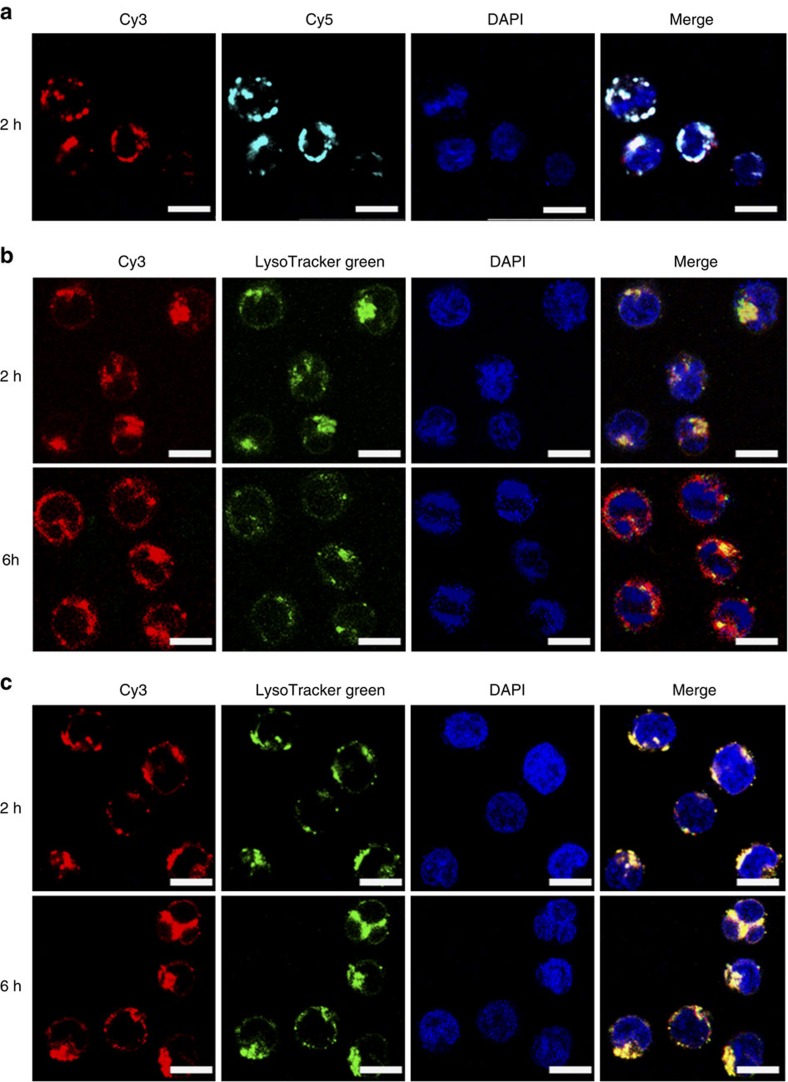
Characterization of endosomal escape. (**a**) Confocal fluorescence microscopic images of CEM cells incubated with Cy3/Cy5-siRNA-ONV. Cy3 and Cy5 were labelled on different siRNA-TRU and self-assembled together to form Cy3/Cy5-siRNA-ONV. The cell nucleus was stained with DAPI. The merge image shows co-localization of both fluorophores in cytoplasm of CEM cells. (**b**–**c**) Co-staining of CEM cells with LysoTracker Green and DAPI after incubated with (**b**) Cy3-siRNA-ONV and (**c**) Cy3-siRNA-ONV-NR for 2 and 6 h. Scale bars, 10 μm.

To confirm the integrity of siRNA-ONV nanostructures when they crossed through cell membrane, Cy3 and Cy5 were labelled on different siRNA-TRUs and the assembled siRNA-ONV was incubated with CEM cells for 2 h. The co-localization experiment showed that the fluorescence from Cy3 and Cy5 appeared nearly in the same place (Fig. 4a). To study the internalization process of siRNA-ONV, co-staining experiment was further performed by staining lysosomes with LysoTracker Green and cell nucleus with 4′,6-diamidino-2-phenylindole (DAPI). Cy3-siRNA-ONV was localized in lysosomes on the same sites after incubation with CEM cells for 2 h by the overlap of red (Cy3-siRNA-ONV) and green (lysosome) fluorescence (Fig. 4b), indicating successful cellular internalization of the Cy3-siRNA-ONV through endocytosis. After incubation with Cy3-siRNA-ONV for 6 h, weaker fluorescence of LysoTracker Green was observed (Fig. 4b), suggesting the spreading of LysoTracker Green from acidic lysosome to neutral cytoplasm^32^,^33^, since the staining of lysosome by the tracker was dependent on the acidicity^34^. These phenomena clearly demonstrated the efficient translocation or escape of Cy3-siRNA-ONV from lysosomes into the cytoplasm due to the rupture of the lysosomal membrane^33^,^35^. During the endocytosis process, the rigid structure of self-assembled ONV nanotube could carry siRNA crossing through cell membrane and protect them from degradation^29^. The successful endosomal escape was also attributed to the rigid linear structure with high aspect ratio of ONV nanotube^36^,^37^,^38^. To demonstrate the rigid structure of ONV prompts endosomal escape process, a control Cy3-siRNA-ONV without rigid structure (Cy3-siRNA-ONV-NR) was synthesized by mixing siRNA-TRU with DNA backbone at a lower ratio of 0.5:1. No fluorescence colour separation was observed after incubating Cy3-siRNA-ONV-NR with CEM cells for 6 h, indicating the failure of endosomal escape process in the absence of rigid structure (Fig. 4c). To further validate the endosomal escape, calcein was used to monitor the stability of lysosomes after Cy3-siRNA-ONV uptake^32^. After CEM cells were treated with the mixture of calcein and Cy3-siRNA-ONV for 6 h, they showed the spreading of calcein to the cytoplasm with strong calcein fluorescence and yellow fluorescence resulted from the overlap of red Cy3 and green calcein fluorescence (Supplementary Fig. 7b), while the cells treated with calcein alone or the mixture of calcein and Cy3-siRNA-ONV-NR only showed the weaker fluorescence inside the endosomes (Supplementary Fig. 7a,c). These results demonstrated the success of endosomal escape of Cy3-siRNA-ONV^39^,^40^. To evaluate the particular internalization pathway of the Cy3-siRNA-ONV into the cells, a series of inhibitors were employed to selectively block different internalization processes (Supplementary Fig. 8). Treatment of the cells with NaN_3_ and sucrose led to a 50–60% reduction in Cy3-siRNA-ONV uptake, suggesting Cy3-siRNA-ONV experienced the clathrin-dependent endocytosis pathway upon entering the cells.

### Characterization of siRNA release

In RNAi, the siRNA strands are generally loaded into the RNA-induced silencing complex, and the antisense strand is separated from sense strand to silence corresponding gene and suppress protein expression^41^. Here siRNA duplexes were released from ONV nanotube in the cytoplasm, which was demonstrated from the PAGE analysis of the mixture of siRNA-ONV and CEM cell lysate (Supplementary Fig. 9a)^42^,^43^, and then incorporated into the RNA-induced silencing complex before it participates in RNAi. This result was also confirmed by fluorescence spectra assay (Supplementary Fig. 9b,c). A strong fluorescent of Cy3 was obtained after over 24-h incubation of SQ-siRNA-ONV with CEM cell lysates, indicating the successful SQ-siRNA-ONV disassembly and siRNA release in cell microenvironment. To further confirm the successful siRNA release in the cytoplasm, SQ-siRNA-ONV was also incubated with CEM cells over 24 h (Supplementary Fig. 9d). These cells showed negligible fluorescence after 6-h incubation, suggesting the integrity of SQ-siRNA-ONV during the endosomal escape process. However, after 24-h incubation, the fluorescence of Cy3 was clearly observed in CEM cells, which demonstrated the successful disassembly of SQ-siRNA-ONV and release of siRNA in cells.

### Cytotoxicity assay

The cytotoxicity was evaluated with a standard 3-(4,5-Dimethylthiazol-2-yl)-2-diphenyltetrazolium bromide (MTT) assay at a series of ONV concentrations, and the results were compared with standard transfection agents, Lipofectamine 2000 (Lipo2000) and Trans IT-TKO (TKO)^44^,^45^. Even at very high concentration of 0.5 μM ONV, CEM cells still kept 96.5% viability (Supplementary Fig. 10). In comparison, the cells treated with Lipo2000 and TKO only exhibited 87.1% and 89.6% viability, respectively. The non-cytotoxicity and good biocompatibility of ONV guaranteed its potential application in clinical intervention.

### Gene silencing assay

**Figure 5:**
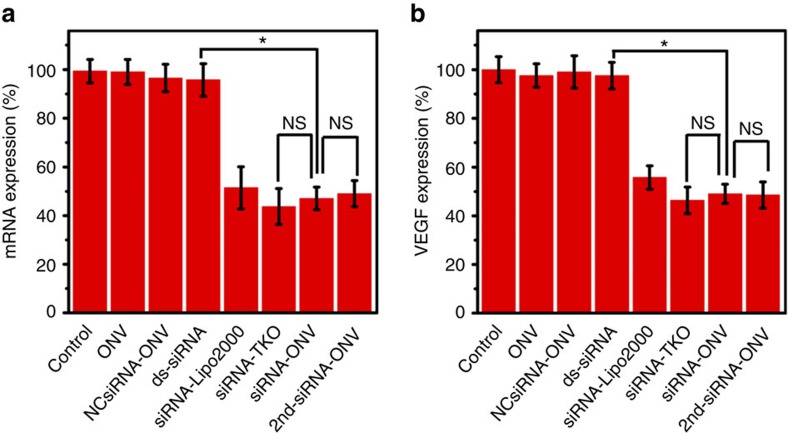
Silencing of target VEGF gene in CEM cells. (**a**) Real-time PCR characterization of mRNA expression for CEM cells incubated with ONV, NCsiRNA-ONV, ds-siRNA, siRNA-Lipo2000, siRNA-TKO, siRNA-ONV and 2nd-siRNA-ONV. (**b**) ELISA characterization of protein secretion levels for CEM cells incubated with ONV, NCsiRNA-ONV, ds-siRNA, siRNA-Lipo2000, siRNA-TKO, siRNA-ONV and 2nd-siRNA-ONV. The data error bars indicate means±s.d. (*n*=3). *P<0.05 (two-tailed Student’s *t*-test).

Vascular endothelial growth factor (VEGF) has been identified as a mitogen and important regulator of angiogenesis, and appeared to be involved in the vascular phase of many different neoplastic diseases. It is a promising approach for human acute lymphoblastic leukaemia treatment through blocking VEGF expression to inhibit tumour vascularization and growth^46^,^47^. To evaluate the therapeutic effect of ‘dual lock-and-key’-controlled siRNA delivery, VEGF was selected as a model therapeutic target and the siRNA-ONV were delivered into target CEM cells against VEGF. After the CEM cells bound with sgc4f and sgc8c aptamers and incubated with 100 nM siRNA-ONV for 48 h, the VEGF messenger RNA (mRNA) and VEGF protein expression levels were determined by real-time PCR assay and enzyme-linked immunosorbent assay (ELISA), and the treatment efficiency was also compared with siRNA-loaded lipo2000 and TKO (siRNA-lipo2000 and siRNA-TKO). A negative control siRNA-ONV (NCsiRNA-ONV) and a second active VEGF siRNA-loaded ONV (2nd-siRNA-ONV) were also synthesized using negative control siRNA and another active siRNA with a different effective sequence to perform the comparison. As observed in Fig. 5, CEM cells treated with just ONV complexes, NCsiRNA-ONV or only ds-siRNA showed little inhibition of VEGF mRNA expression or VEGF protein synthesis. Both the mRNA and protein expression percentage were little decreased compared with CEM cells without any treatment (control). The ‘dual lock-and-key’-controlled delivery approach (siRNA-ONV) demonstrated effective VEGF gene silencing in target cells by inhibiting the VEGF mRNA expression down to 47.1% and VEGF protein production down to 49.1%^48^,^49^,^50^, which was comparable with Lipo2000 mediation transfection, TKO mediation transfection and 2nd-siRNA-ONV.

### Cell apoptosis assay

MTT assay was also used to verify the inhibition effect of siRNA-ONV to CEM cell proliferation. After the CEM cells were incubated with siRNA-ONV, 2nd-siRNA-ONV, siRNA-Lipo2000 or siRNA-TKO for 2 h, their viabilities were compared with those of untreated cells, the cells only treated with ds-siRNA, ONV complex and NCsiRNA-ONV. The ds-siRNA-, ONV complex- or NCsiRNA-ONV-treated cells did not show inhibition effect, and their cell proliferation percentages still remained at ∼100% (Supplementary Fig. 11a). In contrast, siRNA-ONV demonstrated significant inhibition effect on cell proliferation, and the cell proliferation percentage decreased down to 55.7%, at the same level as 2nd-siRNA-ONV, siRNA-Lipo2000 or siRNA-TKO. Moreover, the inhibition percentage of cell proliferation for siRNA-ONV also showed dose-dependent, and the cell proliferation percentage decreased with the increasing siRNA loading (Supplementary Fig. 11b). To further evaluate the therapeutic effect of siRNA-ONV, the cell apoptosis was studied by flow cytometric assay using the Annexin V-fluorescein isothiocyanate (FITC)/propidium iodide (PI) apoptotic kit (Supplementary Fig. 11c). The apoptosis rate for cells treated with siRNA-ONV was 49.3%, comparable with 2nd-siRNA-ONV, siRNA-lipo2000 and siRNA-TKO, and ∼16 times improvement than ds-siRNA. These results demonstrated the feasibility of ‘dual lock-and-key’-controlled siRNA delivery as a promising approach for efficient anticancer therapeutics.

### Therapeutic efficacy under serum condition

To evaluate the therapeutic efficacy of siRNA-ONV and 2nd-siRNA-ONV under serum condition, the gene silencing and cell apoptosis assays were performed under both serum and serum-free conditions. The mRNA expression, protein secretion level and cell proliferation under serum condition showed consistent results with those in reaction buffer (Supplementary Fig. 12), suggesting that the serum did not affect the ‘dual lock-and-key’-controlled siRNA delivery process.

### *In vivo* therapeutic applicability

**Figure 6:**
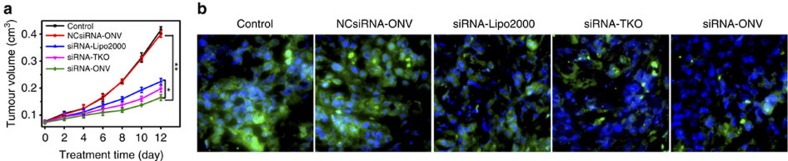
*In vivo* demonstration of therapeutic applicability via target VEGF gene silencing. (**a**) Change of CEM tumour volume after treatment with reaction buffer, NCsiRNA-ONV, siRNA-Lipo2000, siRNA-TKO or siRNA-ONV through intratumoral delivery. The data error bars indicate means±s.d. (*n*=5). *P<0.05, **P<0.01 (two-tailed Student’s *t*-test). (**b**) Expression of VEGF in CEM tumour tissue sections evaluated by immunofluorescence staining.

To demonstrate the *in vivo* inhibition of VEGF expression and the antitumour efficacy of this strategy, the mixture of sgc4f and sgc8c aptamers was first intratumorally injected into mice bearing CEM xenograft tumour for 30 min, and siRNA-ONV, NCsiRNA-ONV, reaction buffer, siRNA-Lipo2000 and siRNA-TKO were then intratumorally injected into these mice, respectively. SiRNA-Lipo2000-, siRNA-TKO- and siRNA-ONV-injected mice presented pronounced inhibition efficacy towards tumour growth compared with the control and NCsiRNA-ONV groups (Fig. 6a). Although the cell experiments for performing siRNA-ONV-, siRNA-Lipo2000- and siRNA-TKO-induced VEGF gene silencing did not show significant difference (Fig. 5), *in vivo* experiments demonstrated slightly higher efficacy (*P*<0.05) of siRNA-ONV than siRNA-Lipo2000 and siRNA-TKO for inhibiting tumour growth (Fig. 6a), suggesting that the high selectivity of ‘dual lock-and-key’ strategy enhanced *in vivo* antitumour activity. Consistently, the immunofluorescence staining of tumour tissue section showed less VEGF protein expression in siRNA-ONV-treated mice than siRNA-Lipo2000-, siRNA-TKO-, NCsiRNA-ONV- and reaction buffer-treated mice (Fig. 6b), demonstrating the downregulated expression level of cytoplasmic VEGF.

## Discussion

In this study, we design a ‘dual lock-and-key’ system for gene interference, which achieves cell-subtype-specific siRNA delivery with high gene silencing efficiency. The siRNA-ONV is prepared by assembling siRNA-TRU repeating units with a DNA backbone produced by RCA, which is used as both the ‘smart key’ and the delivery carrier. The rigid structure of ONV with the high aspect ratio protects siRNA from nuclease degradation (Fig. 2c; Supplementary Fig. 1) when it crosses through the cell membrane, and prompts endosomal escape in the cytoplasm (Fig. 4b). The average length of siRNA-ONV is 0.60±0.15 μm with high loading amount of siRNA up to 84±20 μm (Fig. 2c), offering high delivery efficiency. Two aptamers sgc8c and sgc4f that bind to different cell surface receptors act as ‘dual locks’ sequentially to react with ‘smart key’ siRNA-ONV, thus achieving high specific recognition and siRNA delivery. Both the aptamer ‘locks’ and the self-assembled oligonucleotide nanotube ‘smart key’ can be designed and conveniently synthesized by mature DNA self-assembly technique with high yield and low cost^29^,^30^.

The specificity of ‘dual lock-and-key’-controlled siRNA delivery is verified by using HeLa and Ramos cells as controls. Both CEM and Ramos cells belong to lymphoma cell lines and display the same surface marker sgc4f, and CEM and HeLa cells display the same surface marker sgc8c (Supplementary Fig. 3). These three kinds of cells are indistinguishable for single-receptor-mediated recognition and delivery therapies, therefore multiple cell surface receptors are needed to participate in the delivery process to increase specificity and eliminate off-target toxicities. Through sequential reaction with aptamers sgc8c and sgc4f that, respectively, bind to two different cell membrane receptors, the siRNA is delivered into target CEM cell via the ‘dual lock-and-key’-controlled system (Supplementary Fig. 4). This strategy shows negligible off-target toxicity (Fig. 3), which overcomes potential risk in the conventional single-receptor-targeted delivery system (Supplementary Fig. 6).

The nanomaterials with rigid structure and high aspect ratio such as cerium oxide nanowires and polymer nanoneedles generally exhibit better capability of cellular internalization and endosomal escape, as well as longer circulation times than spherical structure^36^,^38^. Self-assembled DNA nanoribbon is rigid with high aspect ratio and has been confirmed to assist endosomal escape in gene delivery^37^. The siRNA-ONV nanotube has the rigid and tube-like features with high aspect ratio (Fig. 2c) and demonstrates efficient endosomal escape by the co-localization experiment with dyes LysoTracker Green and DAPI (Fig. 4b). In comparison, a control siRNA-ONV without rigid structure (siRNA-ONV-NR) shows the failure of endosomal escape (Fig. 4c), supporting the proposed mechanism of endosomal escape. Compared with other endosomal escape technique based on positively charged polymer^51^, self-assembled DNA nanostructure simplifies synthesis steps and has better biocompatibility, therefore can be used as a universal delivery vehicle for siRNA, drug and protein for cancer therapy.

In conclusion, we present a strategy of the ‘dual lock-and-key’ system with structure switchable smart key for cell-subtype-specific siRNA delivery and gene silencing. SiRNA-ONV is activated on site at CEM cell membrane by reacting with aptamer sgc4f, and subsequently reacts with sgc8c to achieve precise VEGF siRNA delivery. ONV is synthesized by DNA self-assembly technique with a convenient procedure, providing good biocompatibility, high loading efficiency with no adverse side effects. The structure of ONV can protect siRNA from degradation during endocytic process and guarantee efficient release from lysosomes. The self-assembled nanostructure vehicle could be conveniently functionalized and extended as a robust strategy for specific precise delivery of other functional nucleic acids or DNA-binding proteins. This ‘dual lock-and-key’ strategy provides impressive improvement over the single-receptor delivery system by increasing delivery specificity and inhibiting off-target cytotoxicity, therefore is of great importance for siRNA-targeted delivery and tumour therapy.

## Methods

### Reagents

MTT and calcein were purchased from Sigma-Aldrich (USA). Phi29 DNA polymerase, T4 DNA ligase, exonuclease I, exonuclease III and dNTPs were purchased from New England Biolabs Ltd. Annexin V-FITC apoptosis detection kit was purchased from BD Biosciences (USA). Anti-VEGF primary antibody (19003-1-AP) was purchased from Proteintech Group (USA) and 50 times diluted during experiments. FITC-conjugated secondary antibody (111-095-003) was purchased from Jackson ImmunoResearch (USA) and 100 times diluted during experiments. LysoTracker Green and DAPI were purchased from Invitrogen (Carlsbad, CA, USA). SYBR Green I was obtained from Generay Biotech Co., Ltd (China). PBS (pH 7.4) contained 136.7 mM NaCl, 2.7 mM KCl, 8.72 mM Na_2_HPO_4_ and 1.41 mM KH_2_PO_4_. The reaction buffer was prepared using the PBS containing 0.5 mM MgCl_2_ and 50 μM ZnCl_2_, which helped the efficient binding between aptamers and receptors, as well as providing MNAzyme with Zn^2+^ for autocatalytical cleavage of hairpin structure from the ONV. TAMg buffer (1 × ) was composed of 45 mM Tris and 7.6 mM MgCl_2_, with pH adjusted to 8.0 using glacial acetic acid. All other reagents were of analytical grade. All aqueous solutions were prepared using ultrapure water (≥18 MΩ, Milli-Q, Millipore). siRNAs were obtained from GenePharma Co. Ltd (Shanghai, China). The siRNA sequences were as follows: VEGF siRNA, Cy3-sense 5′-Cy3-GGAGUACCCUGAUGAGAUCdTdT-3′, Cy5-sense 5′-Cy5-GGAGUACCCUGAUGAGAUCdTdT-3′, antisense 5′-GAUCUCAUCAGGGUACUCCdTdTCAAAUGGACCAAGGCCAG-3′, 21 base antisense 5′-GAUCUCAUCAGGGUACUCCdTdT-3′; second active VEGF siRNA, sense 5′-ACCUCACCAAGGCCAGCACdTdT-3′, antisense 5′-GUGCUGGCCUUGGUGAGGUdTdTCAAAUGGACCAAGGCCAG-3′; negative control siRNA, sense 5′-UUCUCCGAACGUGUCACGUdTdT-3′ and antisense 5′-ACGUGACACGUUCGGAGAAdTdTCAAAUGGACCAAGGCCA G-3′. All of the DNA were synthesized and purified by Sangon Biotech Co., Ltd (Shanghai, China). Their sequences were listed in Supplementary Table 1.

### Apparatus

Absorption spectra were recorded on an UV-3600 UV–vis-NIR spectrophotometer (Shimadzu Company, Japan). The gel electrophoresis was performed on the DYCP-31BN Electrophoresis Analyser (Liuyi Instrument Company, China) and imaged on a Bio-Rad ChemDoc XRS (Bio-Rad, USA). Fluorescence spectra were measured on an F-7000 spectrofluorophotometer (HITACHI, Japan). Confocal fluorescence imaging of cells was performed on a TCS SP5 confocal laser scanning microscope (Leica, Germany). Flow cytometric analysis was performed on a Coulter FC-500 flow cytometer (Beckman-Coulter). MTT and ELISA assays were carried out on Hitachi/Roche System Cobas 6000 (680, Bio-Rad, USA). Real-time PCR and melting curve measurement were performed on the CFX96 touch real-time PCR detection system (Bio-Rad, USA). AFM imaging was performed under ambient conditions with an Agilent 5500 AFM/SPM system (USA).

### Preparation of circular DNA template

A volume of 4.2 μl of 100 μM phosphorylated linear DNA and 4.2 μl of 100 μM ligation DNA was mixed and annealed at 95 °C for 4 min. After the mixture was slowly cooled to room temperature over 2 h, 1 μl of T4 DNA ligase (400 U μl^−1^), 2 μl of 10 × T4 DNA buffer and 8.6 μl of ultrapure water were added and the solution was incubated at 25 °C for 16 h. The T4 DNA ligase was inactivated by heating at 65 °C for 10 min. A volume of 4 μl of exonuclease I (20 U μl^−1^) and 4 μl of exonuclease III (100 U μl^−1^) was added in the following, the mixture was then incubated at 37 °C for 8 h to degrade ligation DNA. After heating at 80 °C for 15 min to denature the exonuclease I and exonuclease III, the circular DNA template was obtained and stored at 4 °C before use.

### Preparation of DNA backbone

The DNA backbone was prepared by RCA^52^. A volume of 10 μl of 3 μM circular DNA template and 0.5 μl of 100 μM DNA primer was mixed and annealed at 95 °C for 4 min. Then, the mixture was cooled to room temperature over 2 h and incubated with phi29 DNA polymerase (0.2 U μl^−1^), bovine serum albumin (0.4 μg μl^−1^) and dNTPs (0.1 mM) at 37 °C for 5 h in 150 μl of 1 × phi29 reaction buffer. After reaction, the mixture was incubated at 65 °C for 10 min to denature the phi29 DNA polymerase, and then purified by ultrafiltration (100,000 molecular weight cutoff membrane, Millipore) for three times to obtain the DNA backbone. DNA backbone has repeating strand segments with sequences complementary to TRU. The concentration of the repeat unit in DNA backbone was obtained by measuring ultraviolet absorbance at 260 nm and used as the concentration of DNA backbone^29^.

### Preparation of siRNA-ONV

The ONV was prepared based on a modified assembly strategy^28^. In brief, TRU was synthesized by the equimolar mixing of strands C1, C2, V1, R1 and R2 with a final concentration of 5 μM in 1 × TAMg buffer. This mixture was annealed at 95 °C for 4 min and cooled to room temperature over 2 h. The ONV was generated by equimolar mixing the TRU with DNA backbone for 2 h at room temperature. To generate siRNA-modified ONV (siRNA-ONV), the siRNA-TRU was prepared by mixing of strands V1, C1, C2, R1, R2, siRNA and antisense siRNA at a ratio of 1:1:1:1:1:2:2 and assembled with equimolar DNA backbone. The control Cy3-siRNA-ONV without rigid structure (Cy3-siRNA-ONV-NR) was prepared by mixing of siRNA-TRU with DNA backbone at a ratio of 0.5:1.

### PAGE analysis

Native polyacrylamide gel (8%) was prepared using 1 × TBE buffer. The loading sample was prepared by mixing 7 μl DNA sample, 1.5 μl 6 × loading buffer and 1.5 μl UltraPowerTM dye, and placed still for 3 min before injected into polyacrylamide hydrogel. The gel electrophoresis was run at 90 V for 60 min in 1 × TBE buffer and scanned using a Molecular Imager Gel Doc XR.

### AFM imaging

A volume of 6 μl sample was deposited on freshly cleaved mica surface and incubated for 2 min. After wicked away with filter paper, 20 μl ultrapure water was added to wash the mica for two times. The mica was then dried with a nitrogen flow and scanned in tapping mode.

### Serum stability of siRNA-ONV

To verify the serum stability of siRNA-ONV, Cy3-tagged siRNA and BHQ2-tagged R1 and R2 oligonucleotides were used as components to assemble SQ-siRNA-ONV. SQ-ds-siRNA was also synthesized by assembling Cy3-tagged siRNA and BHQ2-tagged R1 and R2, and used as control. Fluorescence from Cy3 was quenched in the beginning due to the close distance between Cy3 and BHQ2. Then, 50 nM of SQ-siRNA-ONV (100 nM fluorophore concentration) and 100 nM of SQ-ds-siRNA were separately diluted with FBS to yield a final concentration of 50 nM fluorophore in 10% FBS 1 × TAMg. The mixture was incubated at 37 °C for 12 h, fluorescence signal recovery was observed with the disassembly of SQ-siRNA-ONV nanotube and release of siRNA. The fluorescence signal recovery was measured every hour with 510 nm excitation and 560 nm emission.

### Thermal stability of siRNA-ONV

The sample for thermal stability assay was prepared by mixing the 19 μl of 1 μM siRNA-ONV sample solution with 1 μl of 20 × SYBR Green I in 0.2 ml PCR tubes (white). Fluorescence data for melting curves were obtained with the real-time PCR detection system (Bio-Rad) during the temperature increase from 30 to 90 °C at 0.2 °C s^−1^ and held for 15 s in every temperature point^53^.

### Cell culture

HeLa cells (KeyGEN Biotech, Nanjing, China) were cultured in DMEM supplemented with 10% FBS, 100 μg ml^−1^ streptomycin and 100 U ml^−1^ penicillin–streptomycin at 37 °C in a humidified incubator containing 5% CO_2_ and 95% air. CCRF-CEM (CCL-119, T-cell line, human acute lymphoblastic leukaemia (ALL)) from KeyGEN Biotech and Ramos (CRL-1596, B-cell line, human Burkitt’s lymphoma) from Cobioer Biosciences (Nanjing, China) were cultured in RPMI 1640 medium containing 10% FBS and 100 U ml^−1^ penicillin–streptomycin at 37 °C in a humidified incubator containing 5% CO_2_ and 95% air. Short tandem repeats (STR) profiling and mycoplasma testing were conducted for each cell line before use. Cell numbers were determined with a Petroff-Hausser cell counter (USA).

### Characterization of aptamers specificity

Sgc8c and sgc4f aptamers were labelled with FAM to characterize the aptamer-binding specificity to different cells. FAM-labelled aptamers were incubated with CEM, Ramos and HeLa cells at 37 °C for 30 min, and then subjected to flow cytometric assay over FL1 channel.

### Confocal fluorescence imaging and flow cytometric assay

A concentration of 50 nM of sgc4f and sgc8c aptamer probes was incubated with 1 × 10^4^ corresponding cells in reaction buffer for 30 min at room temperature. After the cells were centrifuged at 156*g* for 5 min and washed twice with the reaction buffer, 100 nM of Cy3-siRNA-ONV was added and incubated with the cells for 2 h at 37 °C. After washing twice to remove nonbinding Cy3-siRNA-ONV, the fluorescence of cells was visualized from 550 to 610 nm on the confocal laser scanning microscope (CLSM; TCS SP5, Leica, Germany) with the excitation wavelength of 514 nm for Cy3. All images were digitized and analysed with Leica Application Suite Advanced Fluorescence software package. To confirm internalization of Cy3-siRNA-ONV in the CEM cells, the vertical section scanning along the *z* axis was performed. While moving the focal plane in incremental steps from the bottom to the top of the cell within a 12 μm focal plane distance at the *z* position, 20 confocal fluorescence microscopy images were taken and recorded. The flow cytometric assay was performed in the PBS and used FL2 channel.

### Cell co-localization assay

Cy3-labelled siRNA (Cy3-siRNA) and Cy5-labelled siRNA (Cy5-siRNA) were self-assembled with other DNA strand component to form siRNA-TRU, respectively, and the resulting Cy3-siRNA-TRU and Cy5-siRNA-TRU were mixed with equimolar DNA backbone to generate the Cy3/Cy5-siRNA-ONV. After loading with Cy3/Cy5-siRNA-ONV, 5.0 μg ml^−1^ DAPI was added in CEM cells and incubated 15 min for imaging. DAPI was excited with a violet 405-nm laser diode and the emission was collected from 450 to 500 nm. Cy3 was excited at 543 nm with a HeNe 543 laser and the emissions were collected from 560 to 620 nm. Cy5 was excited at 633 nm with a HeNe 633 laser and the emissions were collected from 650 to 700 nm.

### Endosomal escape of siRNA-ONV

To visualize the co-localization of internalized siRNA-ONV with endosomal compartments, the aptamer-bound CEM cells were incubated with Cy3-siRNA-ONV and Cy3-siRNA-ONV without rigid structure (Cy3-siRNA-ONV-NR) at siRNA concentration of 200 nM for 2 or 6 h at 37 °C. Then, the cells were washed with PBS and stained with 100 nM of LysoTracker Green and 5 μg ml^−1^ of DAPI for 15 min for imaging. LysoTracker Green was excited at 488 nm with an argon ion laser and the emission was collected from 505 to 535 nm.

The endosomal escape was further demonstrated with calcein release assay by incubating the aptamer-bound CEM cells with the mixture of 25 μM calcein and Cy3-siRNA-ONV or Cy3-siRNA-ONV-NR for 6 h at 37 °C and then washing these cells with PBS. Calcein was excited at 488 nm with an argon ion laser and the emission was collected from 500 to 530 nm.

### Endocytosis pathways of siRNA-ONV

The uptake inhibitors of 450 mM sucrose, 200 μg ml^−1^ genistein, 50 μM methyl-β-cyclodextrin (Me-β-CD), 50 nM wortmannin and 10 mM NaN_3_ were preincubated with CEM cells for 30 min to inhibit clathrin, caveolae, lipid raft, macropinocytosis and energy-dependent endocytosis, respectively^37^,^54^. Then, the cells were loaded with Cy3-siRNA-ONV and the inhibitors were maintained throughout the experiments. After that, flow cytometric analyses were performed to determine the uptake amount of Cy3-siRNA-ONV.

### Release of siRNA

siRNA release process was first confirmed in cell lysates. A total of 5 × 10^7^ cells were washed twice with cold PBS (pH 7.4) and resuspended in 200 μl of hypotonic lysis buffer containing 20 mM HEPES-KOH, pH 7.4, 10 mM KOAc, 1.5 mM Mg(OAc)_2_, 5 mM dithiothreitol, 0.1% Tween-20 and 1 × EDTA-free protease inhibitor cocktail. The mixture was incubated for 10 min on ice and centrifuged at 35,200*g* and 4 °C for 20 min. The supernatant was collected and diluted to 200 μl as cell lysates for detection. A concentration of 50 nM SQ-siRNA-ONV with 100 nM fluorophore concentration was added in cell lysates or 1 × TAMg buffer. The mixture was incubated at 37 °C for 24 h to measure the fluorescence signal with 510-nm excitation and 560-nm emission. For PAGE assay, 4 μl cell lysate was incubated with 4 μl siRNA-ONV at 37 °C for 1 h and subsequently resolved on a 8% native polyacrylamide gel as described above.

To characterize the intracellular release of siRNA from siRNA-ONV, the SQ-siRNA-ONV was incubated with CEM cells for 24 h, and the fluorescence recovery from released Cy3-siRNA after SQ-siRNA-ONV disassembly was imaged with CLSM.

### MTT assay

MTT assay was carried out to investigate the cytotoxicity of ONV. CEM cells (5 × 10^5^ per well) were seeded into two 96-well plates in 200 μl reaction buffer containing 50 nM of sgc4f and sgc8c aptamers for 30 min. Then, the cells were washed with PBS and incubated with serial concentrations of the ONV for 2 h. The results were compared with commercial transfection agent, 0.4 μl of Lipo2000 and TKO. Cells incubated with PBS served as control. The cells were washed twice with PBS buffer in the following and 50 μl of 5 mg ml^−1^ MTT solution was added and incubated for 4 h. After removing medium, 150 μl of dimethylsulphoxide was added to dissolve the formazan crystals precipitates. After shaking the cell plate for 15 min, the optical density at a wavelength of 490 nm was measured with a Bio-Rad microplate reader. The relative cell viability (%) was calculated by (*A*_test_/*A*_control_) × 100. Negative control siRNA-ONV (NCsiRNA-ONV) was synthesized by using negative control siRNA instead of VEGF siRNA for cell proliferation assay. NCsiRNA-ONV, ds-siRNA, siRNA-Lipo2000, siRNA-TKO and siRNA-ONV at siRNA concentration of 200 nM or equivalent ONV concentration of 100 nM were incubated with CEM cells for 2 h. After siRNA was transfected into CEM cells, fresh culture medium containing 10% FBS was added and incubated with cells for 48 h. Then, the cell proliferation was evaluated with the MTT assay.

### Gene silencing assay

CEM cells were seeded into a 24-well plate at 5 × 10^5^ cells per well and incubated with 50 nM of sgc8c and sgc4f in 200 μl reaction buffer for 30 min. After the cells were washed with PBS, 100 nM of siRNA-ONV was added and incubated for 2 h at 37 °C. Meanwhile, ONV, NCsiRNA-ONV, 2nd-siRNA-ONV, ds-siRNA, siRNA-Lipo2000 and siRNA-TKO at siRNA concentration of 200 nM or equivalent ONV concentration of 100 nM were incubated with CEM cells for 2 h as controls. After centrifugation to precipitate cells, the supernatant medium was replaced by a fresh culture medium containing 10% FBS and further cultured for 48 h. The supernatant of culture medium was collected and analysed by ELISA to quantify VEGF secreted from the cells. Total RNAs from the transfected CEM cells were extracted using the Trizol reagent (Invitrogen), and complementary DNA was generated using the PrimeScriptRT reagent kit (Takara). The synthesized complementary DNA was then run on the CFX96 real-time PCR detection system (Bio-Rad, USA) to calculate VEGF mRNA level of cellular.

### Cell apoptosis experiments

A total of 5.0 × 10^5^ CEM cells were seeded in a 24-well plate in 200 μl reaction buffer containing 50 nM sgc8c and 50 nM sgc4f for 30 min at room temperature. The cells were washed with PBS in the following and incubated with 100 nM of siRNA-ONV for 2 h. The cells were also incubated with ONV, NCsiRNA-ONV, 2nd-siRNA-ONV, ds-siRNA, siRNA-Lipo2000 and siRNA-TKO at siRNA concentration of 200 nM or equivalent ONV concentration of 100 nM under the same condition as the controls. After centrifugation of cells, the supernatant medium were replaced with fresh culture medium containing 10% FBS and further incubated for 48 h. The resulting cells were collected and stained with the mixture of 5.0 μl Annexin V-FITC and 5.0 μl propidium iodide for 15 min, and then imaged with flow cytometry over FL1 (Annexin V-FITC) and FL3 (PI) channels.

### Therapeutic efficacy assay under serum condition

To characterize the therapeutic efficacy of siRNA-ONV and 2nd-siRNA-ONV under serum condition, the internalization of siRNA-ONV and 2nd-siRNA-ONV in CEM cells was performed in fresh culture medium containing 10% FBS, 0.5 mM MgCl_2_ and 50 μM ZnCl_2_, and then the resulting cells were evaluated with gene silencing and cell apoptosis assays.

### *In vivo* antitumour efficacy

Specific pathogen-free female BALB/c nude mice, 5–6 weeks of age, were purchased from Shanghai Laboratory Animal Center, Chinese Academy of Sciences, and bred in an axenic environment. All animal procedures were in accordance with Institutional Animal Use And Care Regulations approved by the Model Animal Research Center of Nanjing University (MARC), which allowed the maximal diameter (length) of every tumour should not exceed 20 mm or the volume of every tumour should be <2.0 cm^3^. To establish a CEM tumour model, 1.0 × 10^6^ CEM cells were subcutaneous injected into the selected position of the nude mice. When the tumour volumes reached to 75 mm^3^, the tumour-bearing mice were randomly divided into five groups and intratumorally injected with 50 μl reaction buffer containing 200 pmol sgc8c and 200 pmol sgc4f, and subsequently injected with 50 μl reaction buffer (control), NCsiRNA-ONV, siRNA-ONV, siRNA-Lipo2000 or siRNA-TKO at a dose of 500 pmol siRNA per mouse after 30 min. The injections were performed every other day for five times. The tumour sizes were measured every 2 days by a digital caliper for 12 days, and the tumour volumes were calculated as *V*=(*L* × *W*^2^)/2, where *L* and *W* were the length and width of the tumour, respectively. Four days after the final injection, tumours were collected, washed by saline thrice, fixed in the 10% neutral buffered formalin, processed routinely into paraffin, sectioned at 4 μm and incubated with anti-VEGF primary antibody at 37 °C for 2 h. The tissue sections were washed with saline thrice, stained with FITC-conjugated secondary antibody and the nuclei were counterstained with DAPI. The stained tissue sections were observed with CLSM.

### Data availability

The authors declare that the data supporting the findings of this study are available within the article and its Supplementary Information files or from the author on request.

## Additional information

**How to cite this article:** Ren, K. *et al*. A DNA dual lock-and-key strategy for cell-subtype-specific siRNA delivery. *Nat. Commun.* **7,** 13580 doi: 10.1038/ncomms13580 (2016).

**Publisher’s note**: Springer Nature remains neutral with regard to jurisdictional claims in published maps and institutional affiliations.

## Supplementary Material

Supplementary Information Supplementary Figures 1-12 and Supplementary Table 1.

Peer Review

## References

[b1] Yu D. B. . Single-stranded RNAs use RNAi to potently and allele-selectively inhibit mutant huntingtin expression. Cell 150, 895–908 (2012). 22939619 10.1016/j.cell.2012.08.002 PMC3444165

[b2] Lima W. F. . Single-stranded siRNAs activate RNAi in animals. Cell 150, 883–894 (2012). 22939618 10.1016/j.cell.2012.08.014

[b3] Kanasty R., Dorkin J. R., Vegas A. & Anderson D. Delivery materials for siRNA therapeutics. Nat. Mater. 12, 967–977 (2013). 24150415 10.1038/nmat3765

[b4] Petros R. A. & DeSimone J. M. Strategies in the design of nanoparticles for therapeutic applications. Nat. Rev. Drug Discov. 9, 615–627 (2010). 20616808 10.1038/nrd2591

[b5] Kanasty R. L., Whitehead K. A., Vegas A. J. & Anderson D. G. Action and reaction: the biological response to siRNA and its delivery vehicles. Mol. Ther. 20, 513–524 (2012). 22252451 10.1038/mt.2011.294 PMC3293611

[b6] Lu H. . Site-specific antibody−polymer conjugates for siRNA delivery. J. Am. Chem. Soc. 135, 13885–13891 (2013). 23924037 10.1021/ja4059525 PMC3955097

[b7] Song E. . Antibody mediated in vivo delivery of small interfering RNAs via cell-surface receptors. Nat. Biotechnol. 23, 709–717 (2005). 15908939 10.1038/nbt1101

[b8] Yao Y. D. . Targeted delivery of PLK1-siRNA by scFv suppresses her2^+^ breast cancer growth and metastasis. Sci. Transl. Med. 4, 130ra48 (2012). 10.1126/scitranslmed.3003601 22517885

[b9] McNamara J. O. II . Cell type–specific delivery of siRNAs with aptamer-siRNA chimeras. Nat. Biotechnol. 24, 1005–1015 (2006). 16823371 10.1038/nbt1223

[b10] Dassie J. P. . Systemic administration of optimized aptamer-siRNA chimeras promotes regression of PSMA-expressing tumors. Nat. Biotechnol. 27, 839–846 (2009). 19701187 10.1038/nbt.1560 PMC2791695

[b11] Zhou H., Jiao P., Yang L., Li X. & Yan B. Enhancing cell recognition by scrutinizing cell surfaces with a nanoparticle array. J. Am. Chem. Soc. 133, 680–682 (2011). 21182273 10.1021/ja108527y

[b12] Kluza E. . Synergistic targeting of α_v_ß_3_ integrin and galectin-1 with heteromultivalent paramagnetic liposomes for combined MR imaging and treatment of angiogenesis. Nano Lett. 10, 52–58 (2010). 19968235 10.1021/nl902659g

[b13] Rudchenko M. . Autonomous molecular cascades for evaluation of cell surfaces. Nat. Nanotechnol. 8, 580–586 (2013). 23892986 10.1038/nnano.2013.142 PMC3776593

[b14] Douglas S. M., Bachelet I. & Church G. M. A logic-gated nanorobot for targeted transport of molecular payloads. Science 335, 831–834 (2012). 22344439 10.1126/science.1214081

[b15] You M. X. . DNA “nano-claw”: logic-based autonomous cancer targeting and therapy. J. Am. Chem. Soc. 136, 1256–1259 (2014). 24367989 10.1021/ja4114903 PMC3935767

[b16] You M. X., Zhu G. Z., Chen T., Donovan M. J. & Tan W. H. Programmable and multiparameter DNA-based logic platform for cancer recognition and targeted therapy. J. Am. Chem. Soc. 137, 667–674 (2015). 25361164 10.1021/ja509263k PMC4308741

[b17] Yan M. . Single siRNA nanocapsules for enhanced RNAi delivery. J. Am. Chem. Soc. 134, 13542–13545 (2012). 22866878 10.1021/ja304649a PMC4318836

[b18] Calabrese C. M. . Biocompatible infinite-coordination-polymer nanoparticle–nucleic-acid conjugates for antisense gene regulation. Angew. Chem. Int. Ed. 54, 476–480 (2015). 10.1002/anie.201407946 PMC4314394 25393766

[b19] Xu X. Y. . Enhancing tumor cell response to chemotherapy through nanoparticle-mediated codelivery of siRNA and cisplatin prodrug. Proc. Natl Acad. Sci. USA 110, 18638–18643 (2013). 24167294 10.1073/pnas.1303958110 PMC3832000

[b20] Kim H. S. . *In vitro* and *in vivo* gene-transferring characteristics of novel cationic lipids, DMKD (O,O′-dimyristyl-N-lysyl aspartate) and DMKE (O,O′-dimyristyl-N-lysyl glutamate). J. Controlled Release 115, 234–241 (2006). 10.1016/j.jconrel.2006.08.003 16989919

[b21] Dong H. F. . Target-cell-specific delivery, imaging, and detection of intracellular microRNA with a multifunctional SnO_2_ nanoprobe. Angew. Chem. Int. Ed. 51, 4607–4612 (2012). 10.1002/anie.201108302 22473624

[b22] Chen Y. J., Groves B., Muscat R. A. & Seelig G. DNA nanotechnology from the test tube to the cell. Nat. Nanotechnol. 10, 748–760 (2015). 26329111 10.1038/nnano.2015.195

[b23] Pei H., Zuo X. L., Zhu D., Huang Q. & Fan C. H. Functional DNA nanostructures for theranostic applications. Accounts Chem. Res. 47, 550–559 (2014). 10.1021/ar400195t 24380626

[b24] Lee J. B., Hong J., Bonner D. K., Poon Z. Y. & Hammond P. T. Self-assembled RNA interference microsponges for efficient siRNA delivery. Nat. Mater. 11, 316–322 (2012). 22367004 10.1038/nmat3253 PMC3965374

[b25] Lee H. . Molecularly self-assembled nucleic acid nanoparticles for targeted *in vivo* siRNA delivery. Nat. Nanotechnol. 7, 389–393 (2012). 22659608 10.1038/nnano.2012.73 PMC3898745

[b26] Zhu G. Z. . Self-assembled, aptamer-tethered DNA nanotrains for targeted transport of molecular drugs in cancer theranostics. Proc. Natl Acad. Sci. USA 110, 7998–8003 (2013). 23630258 10.1073/pnas.1220817110 PMC3657780

[b27] Pacardo D. B., Ligler F. S. & Gu Z. Programmable nanomedicine: synergistic and sequential drug delivery systems. Nanoscale 7, 3381–3391 (2015). 25631684 10.1039/c4nr07677j

[b28] Hamblin G. D. . Simple design for DNA nanotubes from a minimal set of unmodified strands: rapid, room-temperature assembly and readily tunable structure. ACS Nano 7, 3022–3028 (2013). 23452006 10.1021/nn4006329

[b29] Hamblin G. D., Carneiro K. M. M., Fakhoury J. F., Bujold K. E. & Sleiman H. F. Rolling circle amplification-templated DNA nanotubes show increased stability and cell penetration ability. J. Am. Chem. Soc. 134, 2888–2891 (2012). 22283197 10.1021/ja2107492

[b30] Lau K. L., Hamblin G. D. & Sleiman H. F. Gold nanoparticle 3D-DNA building blocks: high purity preparation and use for modular access to nanoparticle assemblies. Small 10, 660–666 (2014). 24115591 10.1002/smll.201301562

[b31] Wu P. W., Hwang K., Lan T. & Lu Y. A DNAzyme-gold nanoparticle probe for uranyl ion in living cells. J. Am. Chem. Soc. 135, 5254–5257 (2013). 23531046 10.1021/ja400150v PMC3644223

[b32] Martens T. F., Remaut K., Demeester J., De Smedt S. C. & Braeckmans K. Intracellular delivery of nanomaterials: how to catch endosomal escape in the act. Nano Today 9, 344–364 (2014).

[b33] Park S. J., Park W. & Na K. Tumor intracellular-environment responsive materials shielded nano-complexes for highly efficient light-triggered gene delivery without cargo gene damage. Adv. Funct. Mater. 25, 3472–3482 (2015).

[b34] Mo R., Jiang T. Y., DiSanto R., Tai W. Y. & Gu Z. ATP-triggered anticancer drug delivery. Nat. Commun. 5, 3364 (2014). 24618921 10.1038/ncomms4364

[b35] Conde J., Oliva N., Atilano M., Song H. S. & Artzi N. Self-assembled RNA-triple-helix hydrogel scaffold for microRNA modulation in the tumour microenvironment. Nat. Mater. 15, 353–363 (2016). 26641016 10.1038/nmat4497 PMC6594154

[b36] Wang X. Z. . Designed synthesis of CeO_2_ nanorods and nanowires for studying toxicological effects of high aspect ratio nanomaterials. ACS Nano 6, 5366–5380 (2012). 22564147 10.1021/nn3012114 PMC3651271

[b37] Chen G. . Enzymatic synthesis of periodic DNA nanoribbons for intracellular pH sensing and gene silencing. J. Am. Chem. Soc. 137, 3844–3851 (2015). 25622178 10.1021/ja512665z

[b38] Kolhar P., Doshi N. & Mitragotri S. Polymer nanoneedle-mediated intracellular drug delivery. Small 7, 2094–2100 (2011). 21695782 10.1002/smll.201100497

[b39] Hu Y. . Cytosolic delivery of membrane-impermeable molecules in dendritic cells using pH-responsive core-shell nanoparticles. Nano Lett. 7, 3056–3064 (2007). 17887715 10.1021/nl071542i

[b40] Febvay S., Marini D. M., Belcher A. M. & Clapham D. E. Targeted cytosolic delivery of cell-impermeable compounds by nanoparticle-mediated, light-triggered endosome disruption. Nano Lett. 10, 2011–2019 (2010). 10.1021/nl101157z PMC4058846 20446663

[b41] Cuellar T. L. . Systematic evaluation of antibody-mediated siRNA delivery using an industrial platform of THIOMAB–siRNA conjugates. Nucleic Acids Res. 43, 1189–1203 (2015). 25550431 10.1093/nar/gku1362 PMC4333408

[b42] Kwok T. . Reduction of gene expression by a hairpin-loop structured oligodeoxynucleotide: Alternative to siRNA and antisense. Biochim. Biophys. Acta 1790, 1170–1178 (2009). 19505533 10.1016/j.bbagen.2009.05.017

[b43] Asbroek A. L. M. A. T., Groenigen M. V., Nooij M. & Baas F. The involvement of human ribonucleases H1 and H2 in the variation of response of cells to antisense phosphorothioate oligonucleotides. Eur. J. Biochem. 269, 583–592 (2002). 11856317 10.1046/j.0014-2956.2001.02686.x

[b44] Liu X. X. . Adaptive amphiphilic dendrimer-based nanoassemblies as robust and versatile siRNA delivery systems. Angew. Chem. Int. Ed. 53, 11822–11827 (2014). 10.1002/anie.201406764 PMC4485617 25219970

[b45] Zhou J. H. . Systemic administration of combinatorial dsiRNAs via nanoparticles efficiently suppresses HIV-1 infection in humanized mice. Mol. Ther. 12, 2228–2238 (2011). 10.1038/mt.2011.207 PMC3242666 21952167

[b46] Fusetti L. . Human myeloid and lymphoid malignancies in the non-obese diabetic/severe combined immunodeficiency mouse model: frequency of apoptotic cells in solid tumors and efficiency and speed of engraftment correlate with vascular endothelial growth factor production. Cancer Res. 60, 2527–2534 (2000). 10811135

[b47] Avramis I. A., Laug W. E., Sausville E. A. & Avramis V. I. Determination of drug synergism between the tyrosine kinase inhibitors NSC 680410 (adaphostin) and/or STI571 (imatinib mesylate, Gleevec) with cytotoxic drugs against human leukemia cell lines. Cancer Chemother. Pharmacol. 52, 307–318 (2003). 12827297 10.1007/s00280-003-0668-y

[b48] Yang Z. Z., Li J. Q., Wang Z. Z., Dong D. W. & Qi X. R. Tumor-targeting dual peptides-modified cationic liposomes for delivery of siRNA and docetaxel to gliomas. Biomaterials 35, 5226–5239 (2014). 24695093 10.1016/j.biomaterials.2014.03.017

[b49] Han L., Tang C. & Yin C. H. Enhanced antitumor efficacies of multifunctional nanocomplexes through knocking down the barriers for siRNA delivery. Biomaterials 44, 111–121 (2015). 25617131 10.1016/j.biomaterials.2014.12.020

[b50] Li J. J. . Aptamer-functionalized silver nanoclusters-mediated cell type-specific siRNA delivery and tracking. Chem. Sci. 4, 3514–3521 (2013).

[b51] Sun W. J. . Self-assembled DNA nanoclews for the efficient delivery of CRISPR–Cas9 for genome editing. Angew. Chem. Int. Ed. 54, 12029–12033 (2015). 10.1002/anie.201506030 PMC4677991 26310292

[b52] Ge J., Zhang L. L., Liu S. J., Yu R. Q. & Chu X. A highly sensitive target-primed rolling circle amplification (TPRCA) method for fluorescent in situ hybridization detection of microRNA in tumor cells. Anal. Chem. 86, 1808–1815 (2014). 24417222 10.1021/ac403741y

[b53] Ririe K. M., Rasmussen R. P. & Wittwer C. T. Product differentiation by analysis of DNA melting curves during the polymerase chain reaction. Anal. Biochem. 245, 154–160 (1997). 9056205 10.1006/abio.1996.9916

[b54] Liang L. . Single-particle tracking and modulation of cell entry pathways of a tetrahedral DNA nanostructure in live cells. Angew. Chem. Int. Ed. 53, 7879–7884 (2014). 10.1002/anie.201403236 24827912

