# Supplementary material for: A DNA dual lock-and-key strategy for cell-subtype-specific siRNA delivery
Source: Nat Commun. 2016 Nov 24;7:13580. doi: 10.1038/ncomms13580 (PMC5476801; doi:10.1038/ncomms13580)
Supplement: Supplementary Information — Supplementary Figures 1-12 and Supplementary Table 1. [file ncomms13580-s1.pdf]

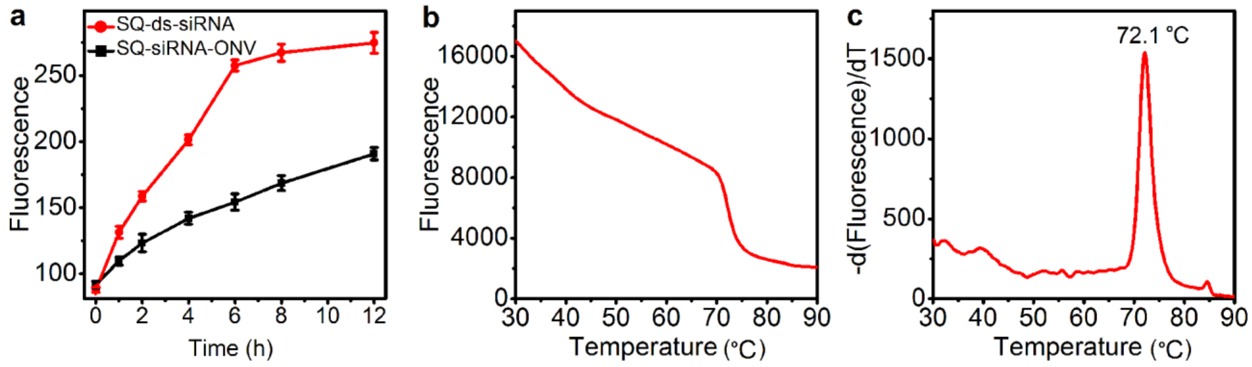

Supplementary Figure 1 | Characterization of siRNA-ONV stability. (**a**) Fluorescence recovery curves of SQ-siRNA-ONV and SQ-ds-siRNA in 1×TAMg buffer containing 10% serum The data error bars indicate means ± SD (n=3). (**b**) Melting curve of siRNA-ONV in thermal stability measurement with real-time PCR. (**c**) Negative first derivative of the melting curve of the siRNA-ONV.

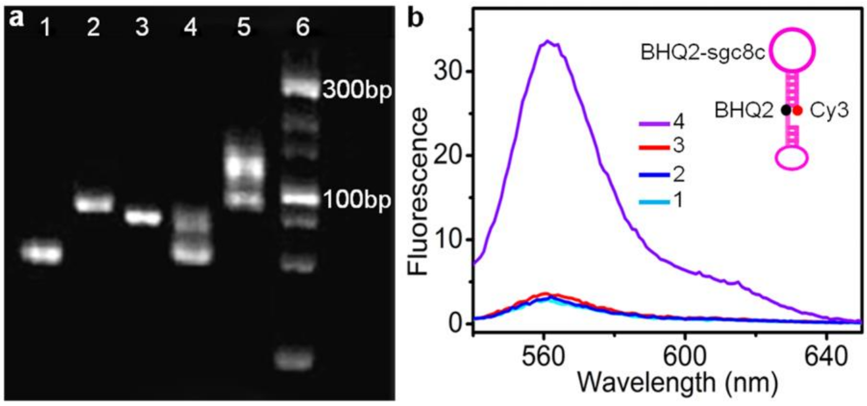

Supplementary Figure 2 | Feasibility of dual parameter controlled "double locks" system. (**a**) PAGE image of (1) DNA primer, (2) sgc4f, (3) sgc8c, (4) mixture of sgc8c and DNA primer, (5) mixture of sgc4f, sgc8c and DNA primer, (6) DNA ladder marker. (**b**) Fluorescence spectra of BHQ2-sgc8c (1) at λ_{ex} of 510 nm after incubated with sgc4f (2), ONV (3), and mixture of sgc4f and ONV (4). Inset in (**b**): illustration of BHQ2-sgc8c.

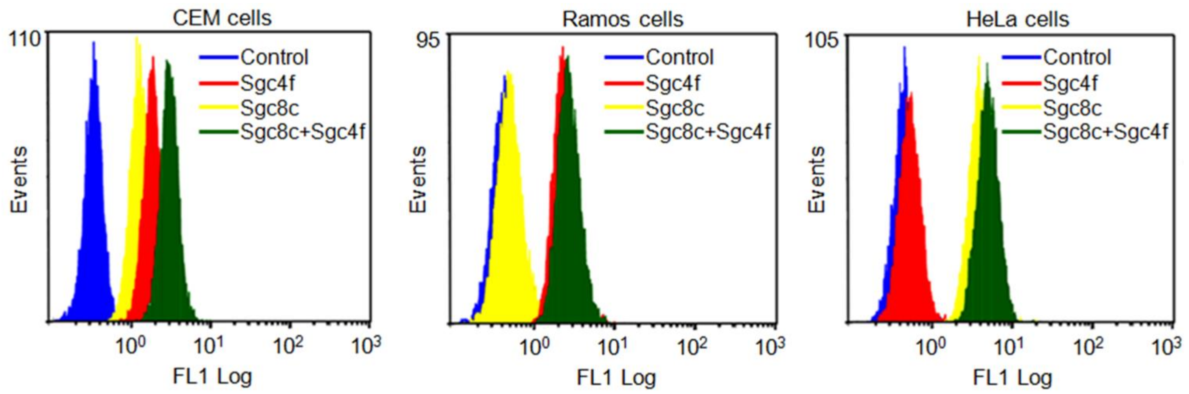

Supplementary Figure 3 | Target recognization of aptamers sgc8c and sgc4f. Flow cytometric assay of CEM, Ramos and HeLa cells incubated with fluorescein dye (FAM) labeled aptamers sgc8c, sgc4f, and mixture of sgc8c and sgc4f aptamers for 30 min.

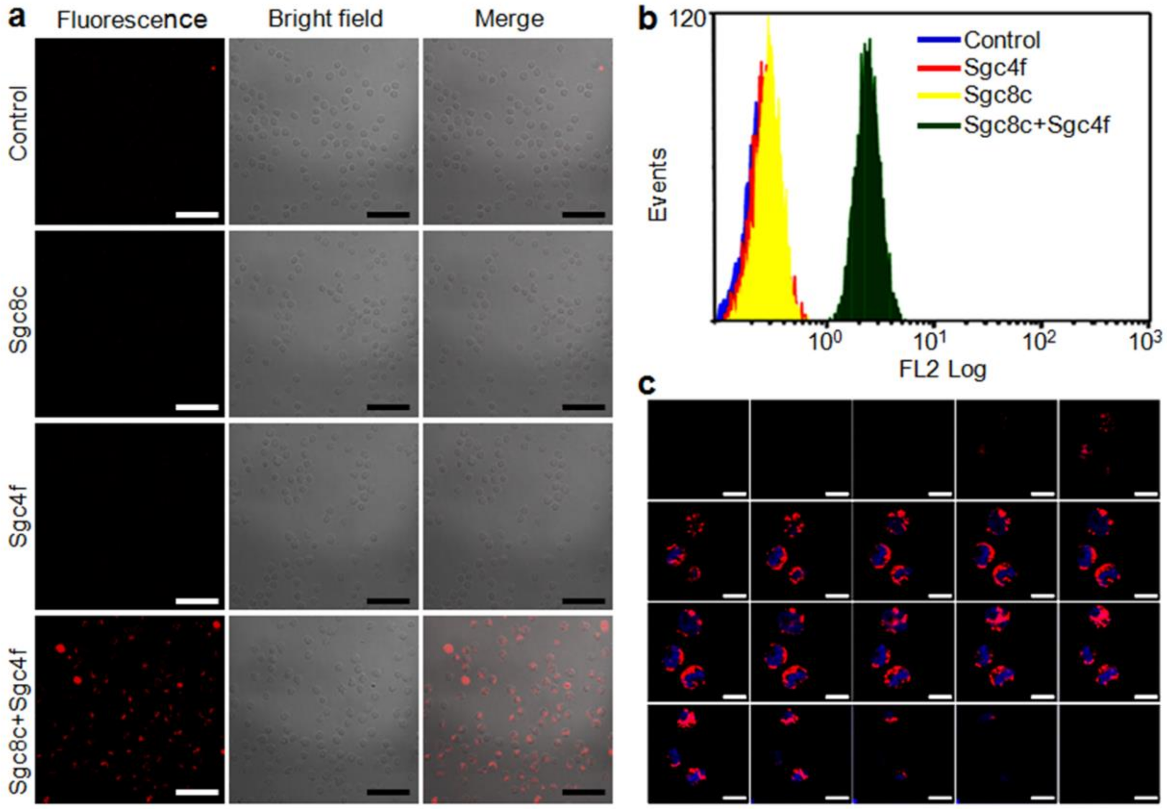

Supplementary Figure 4 | Feasibility of "double locks" system controlled siRNA delivery. (**a**) Confocal fluorescence microscopy images of CEM cells incubated with 100 nM Cy3-siRNA-ONV. CEM cells were pretreated with 50 nM of aptamers sgc8c, sgc4f, and the mixture of sgc8c and sgc4f aptamers. Scale bars: 50 µm. (**b**) Flow cytometric assays of CEM cells incubated with Cy3-siRNA-ONV. CEM cells were pretreated with 50 nM of aptamers sgc8c, sgc4f, and mixture of sgc8c and sgc4f aptamers. (**c**) Z-stack images of CEM cells incubated with 100 nM Cy3-siRNA-ONV. Scale bars: 10 µm.

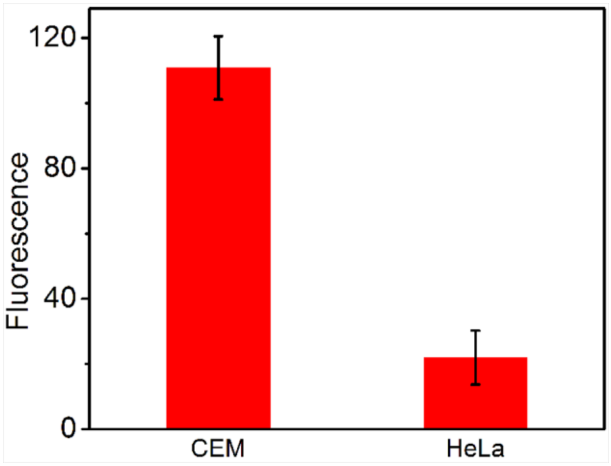

Supplementary Figure 5 | Fluorescence intensity for quantitative analysis of siRNA-ONV internalized in HeLa and CEM cells. Fluorescence intensity obtained from Figure 3 for the mixed HeLa and CEM cells after incubated with 100 nM Cy3-siRNA-ONV for 2 h. The data error bars indicate means ± SD (n=20).

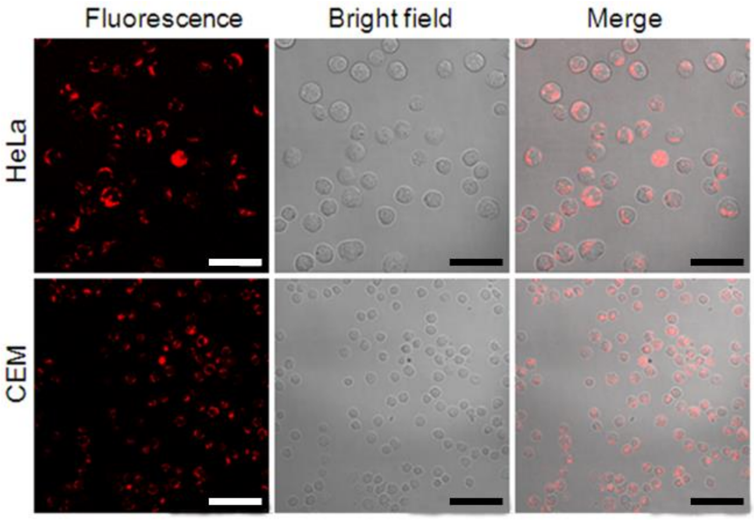

Supplementary Figure 6 | Specificity of single-receptor targeted siRNA delivery. Confocal fluorescence microscopic images of HeLa cells and CEM cells incubated with 100 nM negative control Cy3-siRNA-ONV (S-Cy3-siRNA-ONV). Both cells were pretreated with aptamers sgc4f and sgc8c. Scale bars: 50 μm.

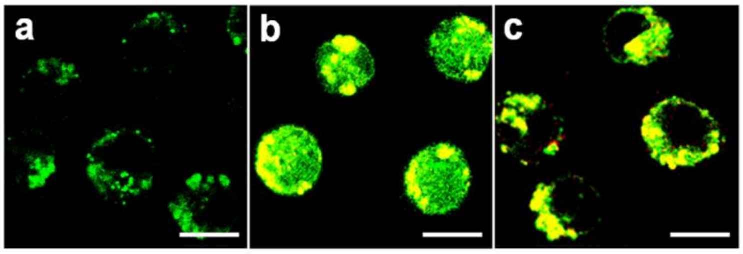

Supplementary Figure 7 | Characterization of endosomal escape with cytoplasmic calcein release assay. Confocal fluorescence microscopic images of CEM cells incubated with (a) 25 μM calcein (green), (b) 25 μM calcein and 100 nM Cy3-siRNA-ONV, and (c) 25 μM calcein and 100 nM Cy3-siRNA-ONV-NR. Scale bars: 10 μm.

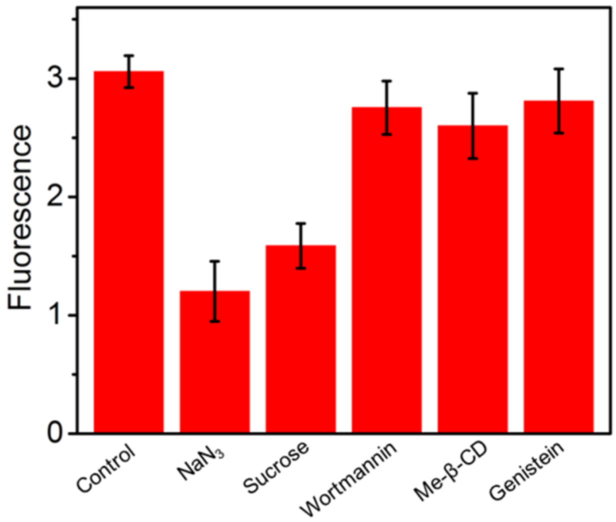

Supplementary Figure 8 | Endocytosis pathways of siRNA-ONV in CEM cells. Uptake inhibition of Cy3-siRNA-ONV in CEM cells preincubated with NaN_3 (10 mM), sucrose (450 mM), wortmannin (50 nM), methyl-β-cyclodextrin (Me-β-CD, 50 μM) and genistein (200 μg mL^{-1}). Fluorescence from Cy3 was measured by flow cytometric assay. The data error bars indicate means ± SD (n=3).

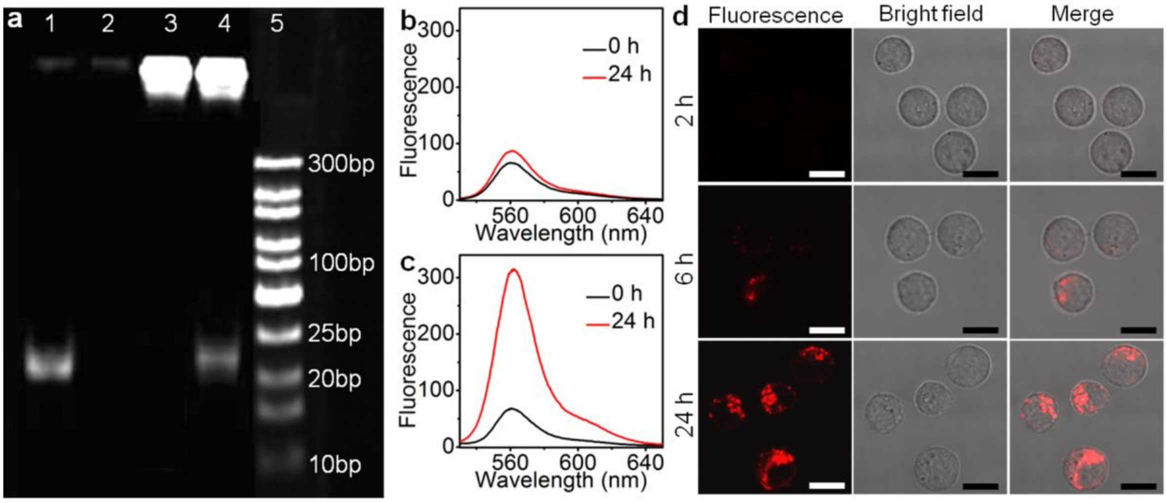

Supplementary Figure 9 | Characterization of siRNA release. (**a**) PAGE image of (1) 21 bp free siRNA, (2) cell lysate, (3) siRNA-ONV, (4) mixture of siRNA-ONV and cell lysate, (5) DNA ladder marker. Fluorescence spectra of SQ-siRNA-ONV nanotube in (**b**) 1×TAMg buffer and (**c**) cell lysate. The fluorescence recovery from Cy3-siRNA was generated by the disassembly of SQ-siRNA-ONV and measured with spectrofluorophotometer. (**d**) Confocal microscopic images of CEM cells loaded with SQ-siRNA-ONV, and incubated for 2, 6 and 24 h. The fluorescence recovery was observed via the disassembly of SQ-siRNA-ONV and release of Cy3-siRNA. Scale bars: 10 μm.

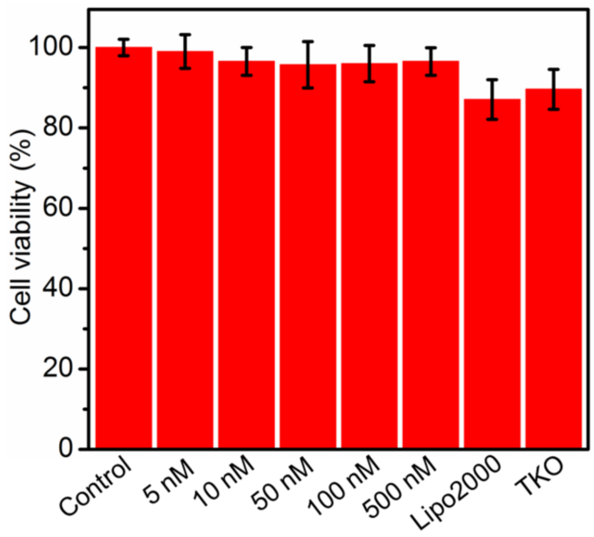

Supplementary Figure 10 | Cytotoxicity assay. Cytotoxicity induced by various concentrations of ONV, Lipo2000 (0.2 μL per 100 μL medium) and TKO (0.2 μL per 100 μL medium) in CEM cells. The data error bars indicate means ± SD (n=3).

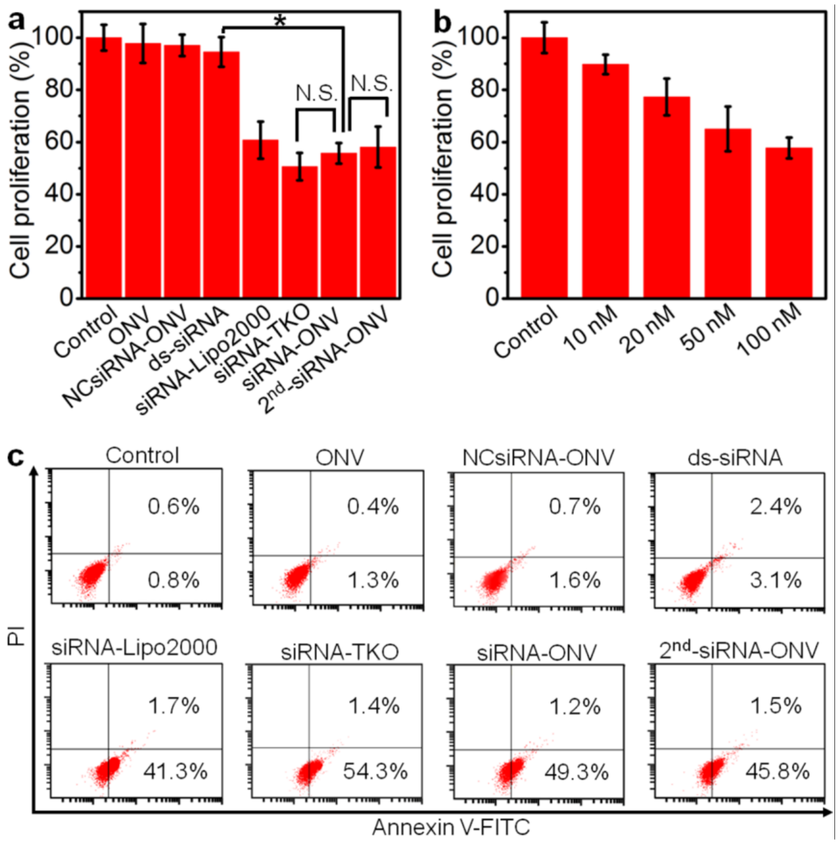

Supplementary Figure 11 | Cell apoptosis assay. Proliferation of CEM cells after incubated with (**a**) ONV (100 nM), NCsiRNA-ONV (100 nM), ds-siRNA (200 nM), siRNA-Lipo2000 (200 nM), siRNA-TKO (200 nM), siRNA-ONV (100 nM) and 2nd-siRNA-ONV (100 nM) respectively, and (**b**) various concentrations of siRNA-ONV. The cell proliferation was tested by the MTT assay, and the data indicate means ± SD (n=3), $*p < 0.05$ (two-tailed Student's t-test). (**c**) Apoptosis of CEM cells after incubated with ONV, NCsiRNA-ONV, ds-siRNA, siRNA-Lipo2000, siRNA-TKO, siRNA-ONV, and 2nd-siRNA-ONV. The cell apoptosis was tested with Annexin V-FITC/PI apoptotic kit.

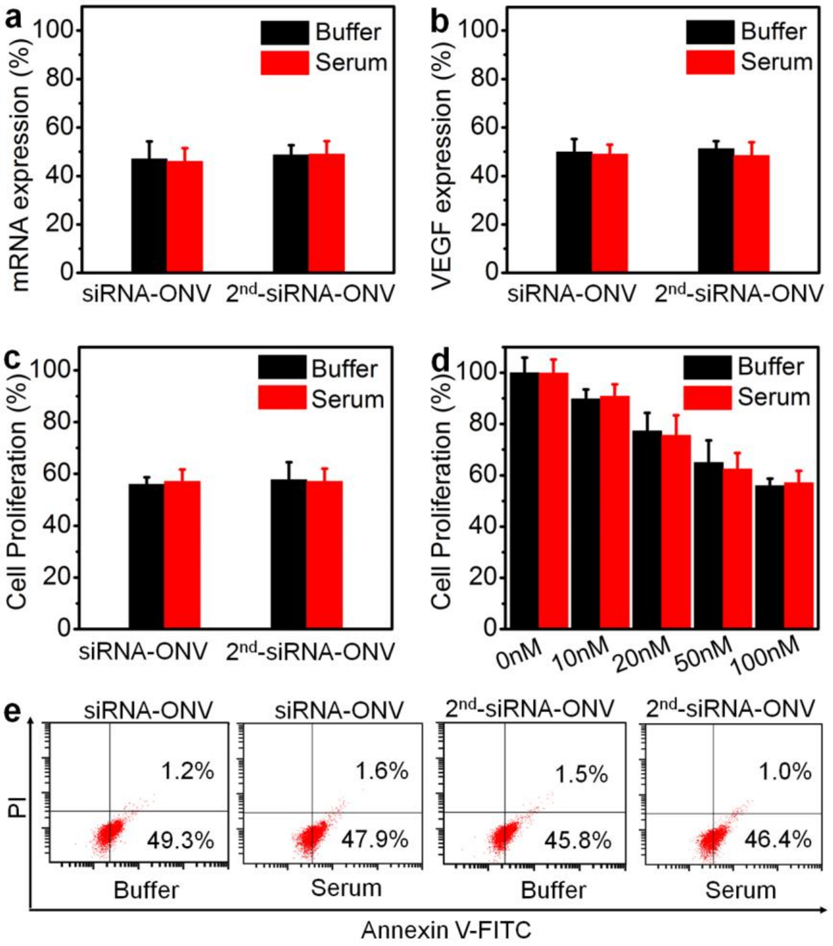

Supplementary Figure 12 | Comparison of therapeutic efficacy under buffer and serum conditions. (**a**) VEGF mRNA expression, (**b**) VEGF protein secretion levels, proliferation of CEM cells after incubated with (**c**) 100 nM siRNA-ONV and 100 nM 2nd-siRNA-ONV and (**d**) various concentrations of siRNA-ONV, and (**e**) apoptosis of CEM cells after incubated with 100 nM siRNA-ONV and 100 nM 2nd-siRNA-ONV. The data error bars indicate means ± SD (n=3).

Supplementary Table 1. Sequences of all oligonucleotides used in this work.

Oligonucleotides	Oligonucleotide sequences
C1	5’-TGTTATCTCCGACGGTACTTCGTACAACGTGCCTCGAATGTAGA<br>GCGTGGCAGGCGGATGTGAAGCAGTTGCACCGGCATTGTC-3’
C2	5’-GTCACTACTAATACACCTGTCGATGAGGTTCCAAGTGTGGATAG<br>CTAGGTAAGACCGCATCTC-3’
R1	5’-AATGCCGGTGCAACTGCTACCAGGTGTATTAGTAGTGACGACT<br>TGCCTGGCCTTGGTCCATTTG-3’
BHQ2-R1	5’-AATGCCGGTGCAACTGCTACCAGGTGTATTAGTAGTGACGACT<br>TGCCTGGCCTTGGTCCATTTG-BHQ2-3’
R2	5’-ATGCGGTCTTACCTAGCTCCAGTACCGTCGGAGATAACAGAGT<br>TGCCTGGCCTTGGTCCATTTG-3’
BHQ2-R2	5’-ATGCGGTCTTACCTAGCTCCAGTACCGTCGGAGATAACAGAGT<br>TGCCTGGCCTTGGTCCATTTG-BHQ2-3’
V1	5’-TCCTAAAGCATGACCTTCCGAACATTCGAGGCACGTTGTACGT<br>CCACACTTGGAACCTCATCGCACATCCGCCTGCCACGCTCTTGTC<br>GGTGAGACGTTTACAGC-3’
DNA primer	5’-**CCACCAC**AATGAAATTGCTATrAGGAAGAGAAGGAG**GTGGTG**<br>**G**AAAAAAAA*GCTGTAAACGTCTCACCG*-3’
Control primer	5’-GAAGGAGGTGGTGGAAAAAAAAGCTGTAAACGTCTCACCG-3’
DNA cycle	5’P-TCCTAAAGCATGACCTTCCGATGT*CGGTGAGACGTTTACAGC*-3’
Connect DNA	5’-TGCTTTAGGAGCTGTAAACG-3’
Sgc4f	5’-ATCACTTATAACGAGTGCGGATGCAAACGCCAGACAGGGGGA<br>CAGGAGATAAGTGAAAAAAAAATTCTCTTC***TCCGAGCCGGTCGA***<br>***A***ATAGCAAT-3’
FAM-sgc4f	5’-ATCACTTATAACGAGTGCGGATGCAAACGCCAGACAGGGGGA<br>CAGGAGATAAGTGAAAAAAAAATTCTCTTC***TCCGAGCCGGTCGA***<br>***A***ATAGCAAT-FAM-3’
Sgc8c	5’-**GGTGGTGGA**AGGACG**TCCACCACC**TCCTTCAAAAAAAAATC<br>TAACTGCTGCGCCGCCGGGAAAATACTGTACGGTTAGA-3’

FAM-sgc8c	5’-FAM-**GGTGGTGGA**AGGACG**TCCACCACC**TCCTTCAAAAAAAAATCTAACTGCTGCGCCGCCGGGAAAATACTGTACGGTTAGA-3’
BHQ2-sgc8c	5’-BHQ2-**GGTGGTGGA**AGGACG**TCCACCACC**(Cy3)TCCTTCAAAAAAAAATCTAACTGCTGCGCCGCCGGGAAAATACTGTACGGTTAGA-3’
VEGF forward	5’-AGGAGGGCAGAATCATCACG-3’
VEGF reverse	5’-CAAGGCCCACAGGGATTTTCT-3’
GAPDH forward	5’-AGGGCTGCTTTTAACTCTGGT-3’
GAPDH reverse	5’-CCCCACTTGATTTTGGAGGGA-3’

The binding regions between DNA primer and sgc4f (or FAM-sgc4f) as well as DNA cycle were shown in underlined and italics. The stem part of hairpin DNA in sgc8c (or FAM-sgc8c, BHQ2-sgc8c) and DNA primer both were shown in bold. The part of MNAzyme catalytic core in sgc4f was shown in italics. The cleavage site for MNAzyme in DNA primer was rA.
