# Supplementary material for: A DNA dual lock-and-key strategy for cell-subtype-specific siRNA delivery
Source: Nat Commun. 2016 Nov 24;7:13580. doi: 10.1038/ncomms13580 (PMC5476801; doi:10.1038/ncomms13580)
Supplement: Peer Review [file ncomms13580-s2.pdf]

**PEER REVIEW FILE**

**Reviewer #1 (Remarks to the Author):**

The MS by Ren et al. reports on a new approach to deliver siRNA to cells. The delivery complex consists of a tube made up of DNA triangles that are assembled by a long DNA strand made by rolling cycle amplification. The siRNAs are hybridized to complementary strands appending from the tube. The delivery vehicle is also connected to a hairpin that can be cleaved by a DNAzyme.

The delivery system is dependent on the presence of two different receptors on the surface of the cells. First these two receptors are targeted by two different DNA aptamers. One of these will cleave the hairpin of the delivery vehicle releasing a strand that can bind to the other receptor that in turn internalize the complex.

The complex has low cytotoxicity and displays an approx. 50% knock down of VEGF expression in CEM cells.

With regard to novelty the DNA structure (triangular tube) was reported earlier (Ref28). Furthermore the two aptamers have been used before in combination for cancer targeting albeit in another systems design (15 and 16).

It is a very interesting study but I have some concerns about the work as listed below:

1) At the end of the MS the authors state that "This study will potentially revolutionize the field of siRNA targeted delivery and meanwhile provide new possibilities for application in precise tumor therapy." I do no believe that. If the system is applied to an animal or even clinically, the DNA aptamers and delivery vehicle are to fragile to survive the two step procedure of adding the aptamers and waiting for them to find the receptors in the organism and subsequently adding the delivery vehicle.

In the experiments the magnesium concentration is much higher that under physiological conditions.

The large DNA tubes would most likely trigger a immunogenic response and it can also be questioned if such large structures would reach the cancer cells.

2) Figure 4. The data shown in this figure should show that endosomal excape is observed for the Cy3-siRNA-ONV after 6h. In 4b after 2h the patterns observed in the images of Cy3 (siRNA-ONV) and Lysotracker Green (that stains the lysosomes) are so unrealistically identical that one must suspect that the authors have problems with the filters and are in fact observing the same dye. After 6h the patterns of Cy3 and the Lysotracker Green stained images are different and the authors claim that is because of endosomal escape. However suddenly the Lysotracker intensity is much lower after 6h (While that is not observed for the reference image in 6c) and I claim that the difference is just a matter of the weak intensity of the Lysotracker image. If one compares the few spots in the Lysotracker Green image after 6h (4c) they are in fact located in the same position as the most intense patterns of the Cy3 dye.

The athors cannot claim anything about lysosomal escape based on these data.

3) Figure 5. The authors compares the knockout of VEGF (mRNA and protein) induced by the siRNA-ONV and siRNA-Lipo2000 and observe that they it is similar of around 50%. Why is the reference siRNA-Lipo2000 knockout so poor here. Normaly much better knockout is observed for that.

4) It is a concern for the selectivity of the system that the siRNA-ONV can diffuse away to other cells after cleavage by Sgc4f. Would it be possible to quantify how large a share of the siRNA-ONV that is activated by the first aptamer is actually internalized.

5) The structures of the aptamers are very poorly shown in Fig 1a

In conclusion this manuscript is not suitable for publication in its current form and only if the authors can carefully address each of the issues addressed above it should be considered for publication.

**Reviewer #2 (Remarks to the Author):**

This report describes a novel design for siRNA targeted delivery and activation which should prove useful for different aptamer siRNA combinations. The work is complete and this reviewer sees no need for additional data.

## To Reviewer 1:

*1. At the end of the MS the authors state that "This study will potentially revolutionize the field of siRNA targeted delivery and meanwhile provide new possibilities for application in precise tumor therapy." I do not believe that. If the system is applied to an animal or even clinically, the DNA aptamers and delivery vehicle are too fragile to survive the two step procedures of adding the aptamers and waiting for them to find the receptors in the organism and subsequently adding the delivery vehicle.*

*In the experiments the magnesium concentration is much higher that under physiological conditions.*

*The large DNA tubes would most likely trigger an immunogenic response and it can also be questioned if such large structures would reach the cancer cells.*

**Response:** Thank you very much for your kind and valuable comments. We agree that it is very challenging to apply this strategy to an animal right now considering the above mentioned limitations, especially for intravenous delivery. To perform *in vivo* demonstration of therapeutic applicability of the proposed strategy, intratumoral delivery method that can help drugs escape from immune clearance, increase local drug concentration, and shorten the time to find the receptors (*J. Am. Chem. Soc.* **2016**, *138*, 704–717; *J. Control. Release* **2015**, *218*, 94–113) has been used to assume the therapeutic effect. The mixture of sgc4f and sgc8c aptamers was firstly injected into CEM xenograft tumor on mice. After 30 min that allowed aptamers bound to CEM cell receptors, siRNA-ONV was injected into CEM xenograft tumor, and the tumor size was continuously measured for 12 days. The immunofluorescence staining was then performed on tumor tissue sections. Compared with control groups treated with reaction buffer injection or commercial transfection reagent Lipo2000 delivery, the treatment with the proposed strategy showed stronger inhibition to the tumor growth, and improved suppression of VEGF expression. The *in vivo* treatment effect with intratumoral delivery indicated the feasibility of aptamer delivery to the receptors to perform the proposed function in the organism, and DNA tubes triggered immunogenic response did not obviously affect the reach of such large structures to the cancer cells on nude mice. The experiment results have been added as Figure 6, and the detailed experiment information has been added in page 21 lines 11-26 and page 29 lines 7-14, and discussed in page 12 lines 11-23.

The reaction buffer used for both cell experiments and *in vivo* intratumoral injection contained 0.5 mM $MgCl_2$. The concentration of magnesium ion was in the physiological

conditions of 0.2-0.8 mM (*Tumor Biol.* **2014**, *35*, 9505–9521). It must be added to help efficient binding between aptamers and receptors.

Although the *in vivo* demonstration of intratumoral injection was successful, there is still a long way from intratumoral delivery to its clinic application. Thus the statement of "This study will potentially revolutionize the field of siRNA targeted delivery and meanwhile provide new possibilities for application in precise tumor therapy" in the original manuscript is not appropriate. According to your opinion, it has been revised as "This 'double locks-smart key' strategy provides impressive improvement over single receptor delivery system by increasing delivery specificity and inhibiting off-target cytotoxicity, therefore is of great importance for siRNA targeted delivery and tumor therapy." in page 14 lines 21-23.

*2. Figure 4. The data shown in this figure should show that endosomal escape is observed for the Cy3-siRNA-ONV after 6h. In 4b after 2 h the patterns observed in the images of Cy3 (siRNA-ONV) and Lysotracker Green (that stains the lysosomes) are so unrealistically identical that one must suspect that the authors have problems with the filters and are in fact observing the same dye. After 6 h the patterns of Cy3 and the Lysotracker Green stained images are different and the authors claim that is because of endosomal escape. However suddenly the Lysotracker intensity is much lower after 6 h (While that is not observed for the reference image in 6c) and I claim that the difference is just a matter of the weak intensity of the Lysotracker image. If one compares the few spots in the Lysotracker Green image after 6 h (4c) they are in fact located in the same position as the most intense patterns of the Cy3 dye. The authors cannot claim anything about Lysosomal escape based on these data.*

**Response:** Thank you very much for the comments. In fact, the patterns in Figure 4b (after 2 h) for the images of Cy3 (siRNA-ONV) and Lysotracker Green (that stains the lysosomes) are different. They are stained with different dyes, and show much different fluorescent wavelength and intensity. To better understand, the intensity ratios of spots **a** to **b** for Cy3 and Lysotracker Green, as shown in followed picture (line 1 in Figure 4b), are compared. The ratio for Cy3 is 0.187, while it is 2.41 for Lysotracker Green. The overlapped patterns of Cy3 and Lysotracker Green result from the fact that Cy3-siRNA-ONV (0.60 ± 0.15 μm, page 5 line 19) occupies most space of lysosome (0.2-1 μm, *J. Am. Chem. Soc.* **2015**, *137*, 3844–3851) at 2 h.

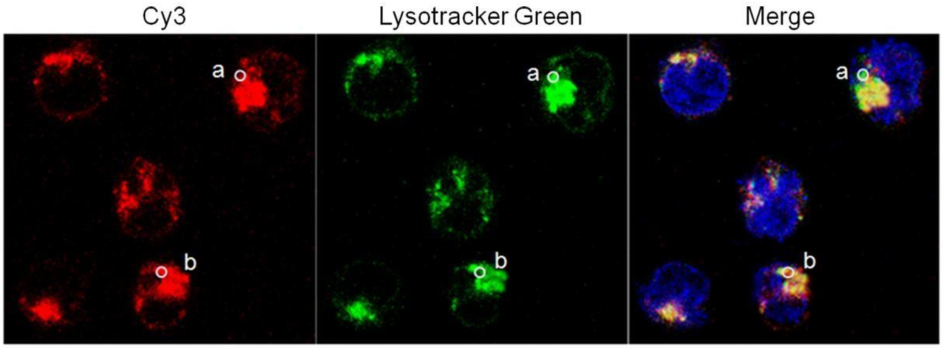


After treated with Cy3-siRNA-ONV for 6 h, the Lysotracker Green intensity is much lower than that at 2 h due to its spreading from acidic lysosome to neutral cytoplasm (*Nano Today* **2014**, *9*, 344–364; *Adv. Funct. Mater.* **2015**, *25*, 3472–3482), which is in agreement with previous reports on endosomal escape (*Nat. Commun.* **2014**, *5*, 3364; *Nat. Mater.* **2016**, *15*, 353–363). Different from Figure 4b, after treated with Cy3-siRNA-ONV-NR for 6 h, the spreading or weaker fluorescence is not observed (Figure 4c), thus the Lysotracker Green does not escape from the acidic lysosome, and it is in fact located in the same position as Cy3. These results demonstrated the validity of the proposed strategy. In revision the related description has been added in page 9 lines 5-8 with four new references as refs 32-35 from page 24 line 21 to page 25 line 7.

To further demonstrate the endosomal escape of Cy3-siRNA-ONV, we also performed the cytoplasmic calcein release assay, and the result has been shown in added Supplementary Figure 7. After CEM cells were treated with the mixture of calcein and Cy3-siRNA-ONV for 6 h, they showed the spreading of calcein to the cytoplasm with strong calcein fluorescence (Supplementary Figure 7b), which indicated the successful endosomal escape of the delivery vehicle (*Nano Lett.* **2007**, 7, 3056–3064; *Nano Lett.* **2010**, *10*, 2211–2219). In contrast, CEM cells treated with calcein alone or the mixture of calcein and Cy3-siRNA-ONV-NR only showed the weaker fluorescence inside the endosomes (Supplementary Figure 7a,c), indicating the failure of endosomal escape in the absence of siRNA-ONV. The detailed information on the experiment has been added in page 19 lines 10-13, and discussed in page 9 lines 16-23 with two new references as refs 39 and 40 in page 25 lines 14-18.

*3. Figure 5. The authors compares the knockout of VEGF (mRNA and protein) induced by the siRNA-ONV and siRNA-lipo2000 and observe that they it is similar of around 50%. Why is the reference siRNA-Lipo2000 knockout so poor here. Normally much better knockout is observed for that.*

**Response:** The therapeutic effect of siRNA is related with its type and dosage. Around 50% knockout of VEGF for siRNA-Lipo2000 is normal considering the concentration of 200 nM for VEGF siRNA, which is consistent with that of around 50% knockout in previous works of Lipo2000 mediated VEGF siRNA transfection (*Biomaterials* **2014**, *35*, 5226–5239;

*Biomaterials* **2015**, *44*, 111–121; *Chem. Sci.* **2013**, *4*, 3514–3521, *et al*). Three new references

have been added as refs 44-46 in page 11 line 18 and page 26 lines 5-11.

*4. It is a concern for the selectivity of the system that the siRNA-ONV can diffuse away to other cells after cleavage by sgc4f. Would it be possible to quantify how large a share of the siRNA-ONV that is activated by the first aptamer is actually internalized.*

**Response:** Thank you very much for the comment. To quantify the percentage of siRNA-ONV activated by sgc4f aptamer to be internalized in CEM cells, we used Cy3-siRNA-ONV to incubate the mixture of CEM cells with both sgc4f and sgc8c acceptors and HeLa cells with only sgc8c acceptor. The result has been added in Figure 3 marked as "HeLa+CEM" (the figure caption has also been modified in page 28 line 13-14), which shows bright fluorescence within CEM cells and little fluorescence within HeLa cells. The fluorescence intensity analysis of the image demonstrates 4.6-fold fluorescence intensity of CEM over HeLa cells, suggesting that 82.1% activated siRNA-ONV is internalized in CEM. The fluorescence intensity analysis has been shown in added Supplementary Figure 5, and the result has been discussed in page 8 lines 3-9. Accordingly, Supplementary Figures 5-9 in original version have been changed as Supplementary Figures 6, and 8-11.

5) *The structures of the aptamers are very poorly shown in Fig 1a.*

**Response:** Thank you very much for your criticism. In fact, there is no aptamer shown in Figure 1a, which only shows the synthesis of triangular rung units (TRU) with seven oligonucleotides of C1, C2, V1, R1, R2, antisense siRNA, and siRNA strands (page 5 lines 5-7). Two kinds of aptamers, sgc8c and sgc4f, are shown in Figure 1c, which are bound on cell surface to act as the "double locks" to control siRNA-ONV delivery. According to your comment, the structures of both aptamers have been redrawn in Figure 1c.

## To Reviewer 2:

This report describes a novel design for siRNA targeted delivery and activation which

should prove useful for different aptamer siRNA combinations. The work is complete and this reviewer sees no need for additional data.

**Response:** Thank you very much for your positive opinion and recommendation. According to the opinions and suggestions of Reviewer 1 and the editor, we have performed in vivo applicability demonstration of the proposed strategy and provided further experimental data to address their criticisms.

**Reviewer #1 (Remarks to the Author):**

The authors have addressed my comments to issue 1 by performing additional animal experiments and by changing the text.

For issue 2 the additional calcein experiments strengthen the arguments by the authors. As I understand it the authors assume that the escape of Cy3-siRNA-ONV also causes the Lysotracker to escape from the endosome. If so it should be clearly explained in the MS.

For issue 5 I am still not happy with Figure 1 (Not only 1a). For example in 1c double stranded DNA is show in at least 4 different ways: 1) in the sgc8c hairpin, straight lines of DNA with orthogonal lines indicating DNA base-pairing, 2) In the "smart key" the end of the red hairpin is just shown as a single line that is opened when the hairpin is cleaved (ok to reduce the complexity of the rest of the siRNA construct since it is shown above), 3) base pairing between sgc4f and the key is just shown by aligning the curved lines, 4) the hybridization between sgc8c and the opened key is now shown as a strange oval shape with lines to show the base pairing. It has to be shown in a more consistent manner.
It should also be clear from the figure or figure caption that the base at the cleavage site in the smart key is a ribonucleotide.

**Reviewer #3 (Remarks to the Author):**

The manuscript by Ren et al. describes and interesting and novel approach for the targeted delivery of siRNAs. Although extensive work has been performed to demonstrate specific binding, uptake, intracellular release and in vitro and in vivo efficacy, there are still significant gaps to be addressed in order to prove on-target efficacy.

1. One main limitation of this manuscript is the lack of a direct proof for intracellular release of functional siRNA. There are several commercial kits available for miRNA and siRNA quantitation to test for correct processing and release of the siRNA from the nucleic acid particle. Without this additional information it is not possible to discriminate between a general degradation of the particle and the intended cleavage and release of siRNA suitable for RISC recruitment.

2. Currently, the demonstration of in vitro efficacy rests on a single active siRNA. Given the significant sequence space available for siRNA design against VEGF, the authors should include knockdown and survival data for a second active siRNA showing similar effects to the chosen active siRNA.

3. The in vivo application of the system lacks a non-targeting siRNA control to test for off-target effects caused by the intratumoural injection of the formulations. The authors must include at least one control in their in vivo experiments for convincingly suggesting on-target efficacy in vivo.

4. Like many other haematopoietic cell lines, CEM cells are poorly transfectable by Lipofectamine 2000 and related cationic lipids. Thus, it is not a "gold standard" for siRNA delivery to such cell lines and, given its sensitivity towards serum, it sheds further doubt on the in vivo on-target effects of the IT injected complexes, which needs to be addressed by proper non-targeting controls.

5. How does serum affect the cell association, uptake and efficacy of the formulations in tissue culture? So far, the authors seem to have tested the formulations under serum-free conditions. This should be clarified in the text, and if only serum-free conditions have been used, at least the efficacy studies need to be repeated under serum.

**To Reviewer 1:**

1) *The authors have addressed my comments to issue 1 by performing additional animal experiments and by changing the text. For issue 2 the additional calcein experiments strengthen the arguments by the authors. As I understand it the authors assume that the escape of Cy3-siRNA-ONV also causes the Lysotracker to escape from the endosome. If so it should be clearly explained in the MS.*

**Response:** Thank you very much for your kind and valuable comments. Yes, the colocalization experiments with both LysoTracker Green and calcein demonstrated the escape of Cy3-siRNA-ONV from the endosome. The weaker fluorescence of LysoTracker Green suggests the spreading of LysoTracker Green from acidic lysosome to neutral cytoplasm[32,33], since the staining of lysosome by the tracker was dependent on the acidicity[34]. This phenomenon is identical with the spreading of calcein to the cytoplasm due to the rupture of the lysosomal membrane.[33,35] The related explanation has been added in page 9 lines 6-10.

2) *For issue 5 I am still not happy with Figure 1 (Not only 1a). For example in 1c double stranded DNA is show in at least 4 different ways: 1) in the sgc8c hairpin, straight lines of DNA with orthogonal lines indicating DNA base-pairing, 2) In the "smart key" the end of the red hairpin is just shown as a single line that is opened when the hairpin is cleaved (ok to reduce the complexity of the rest of the siRNA construct since it is shown above), 3) base pairing between sgc4f and the key is just shown by aligning the curved lines, 4) the hybridization between sgc8c and the opened key is now shown as a strange oval shape with lines to show the base pairing. It has to be shown in a more consistent manner.*

*It should also be clear from the figure or figure caption that the base at the cleavage site in the smart key is a ribonucleotide.*

**Response:** Thanks a lot for your kind advice. Using straight lines of DNA with orthogonal lines to indicate DNA base-pairing is better. Thus we have revised the hybridization scheme between sgc8c and the opened key (way 4) in Figure 1c, consistent with the sgc8c hairpin (way 1). Way 2 is really to reduce the complexity. Way 3 shows the scheme of cleaved "smart key", not the base pairing, which is noted as "On-site autocleavage of siRNA-ONV" in Figure 1c. The cleavage of smart key leads to the departure of smart key from sgc4f. According to your comment, the base "rA" at the cleavage site in the smart key has been illustrated in Figure 1b.

**To Reviewer 3:**

1) *One main limitation of this manuscript is the lack of a direct proof for intracellular release of functional siRNA. There are several commercial kits available for miRNA and siRNA quantitation to test for correct processing and release of the siRNA from the nucleic acid particle. Without this additional information it is not possible to discriminate between a general degradation of the particle and the intended cleavage and release of siRNA suitable for RISC recruitment.*

**Response:** Thank you very much for your kind and valuable comments. Although several commercial kits available for miRNA and siRNA quantitation, it is difficult for us to discriminate conjugated siRNA and free siRNA. To provide a direct proof for intracellular release of functional siRNA, we used PAGE analysis to verify the release of the siRNA from the nucleic acid particle (siRNA-ONV). After 1-h incubation of siRNA-ONV with CEM cell lysate, only one new band appeared at the same position as the free siRNA (21 bp), suggesting the intended cleavage and release of integrated siRNA from siRNA-ONV. This experiment also excludes the possible degradation of the particle, because no random band with different bp numbers is observed. The PAGE analysis has been added as Supplementary Figure 9a, and the detailed experiment information has been added in page 20 lines 19-21, and discussed in page 10 lines 9-10.

2. *Currently, the demonstration of in vitro efficacy rests on a single active siRNA. Given the significant sequence space available for siRNA design against VEGF, the authors should include knockdown and survival data for a second active siRNA showing similar effects to the chosen active siRNA.*

**Response:** Thank you very much for the valuable suggestion. The knockdown and survival data for the second active siRNA have been added in Figure 5 and Supplementary Figure 11, which show similar effects to the chosen active siRNA. The detailed information on the experiment has been added in page 21 lines 14 and 25, and discussed in page 11 lines 13, 22 and page 12 lines 1, 7, 13. The figure legend to Figure 5 has also been modified in page 30 lines 3 and 5.

3. *The in vivo application of the system lacks a non-targeting siRNA control to test for off-target effects caused by the intratumoural injection of the formulations. The authors must include at least one control in their in vivo experiments for convincingly suggesting on-target*

*efficacy in vivo.*

**Response:** The non-targeting siRNA control for examining the off-target effects of intratumoural injection has been added in Figure 6, which do not show the inhibition effect towards tumor growth, indicating the in vivo on-target efficacy of the chosen active siRNA. The detailed information on the experiment has been added in page 22 line 16, and discussed in page 13 lines 2-12. The figure legend to Figure 6 has also been modified in page 30 line 9 and line 12.

4. *Like many other haematopoietic cell lines, CEM cells are poorly transfectable by Lipofectamine 2000 and related cationic lipids. Thus, it is not a "gold standard" for siRNA delivery to such cell lines and, given its sensitivity towards serum, it sheds further doubt on the in vivo on-target effects of the IT injected complexes, which needs to be addressed by proper non-targeting controls.*

**Response:** Thank you very much for bring the concern. To exclude the doubt caused by Lipofectamine 2000, we have supplemented some experiments as proper non-targeting control by using a commercial transfection carrier Trans IT-TKO (TKO) to deliver siRNA into CEM cells, according to the reports in *Angew. Chem. Int. Ed.* **2014**, *53*, 11822–11827 and *Mol. Ther.* **2011**, *12*, 2228–2238. The VEGF gene silencing results of the control have been added in Figure 5 with legend modification in page 30 lines 3 and 5, and the cytotoxicity and cell apoptosis assays have been added in Supplementary Figure 10 and Supplementary Figure 11. These cell experiments show similar transfection efficiency to that of siRNA-ONV. Moreover, the in vivo experiments demonstrated slightly higher efficacy ($p < 0.05$) of siRNA-ONV than siRNA-TKO for inhibiting tumor growth and VEGF expression, which have been shown in Figure 6, suggesting that the high selectivity of “double locks-smart key” strategy enhances in vivo antitumor activity. The detailed information on the experiment has been added in page 21 lines 2, 9, 15 and 25, and page 22 line 17, and discussed in page 10 line 22, page 11 lines 1, 12 and 22, page 12 lines 2, 8, 13 and page 13 lines 2, 3, 6, 8 and 11 with two new references as refs 42 and 43 in page 26.

5. *How does serum affect the cell association, uptake and efficacy of the formulations in tissue culture? So far, the authors seem to have tested the formulations under serum-free conditions. This should be clarified in the text, and if only serum-free conditions have been used, at least the efficacy studies need to be repeated under serum.*

**Response:** According to your suggestion, we have tested the therapeutic efficacy of

siRNA-ONV under serum condition and added the results in Supplementary Figure 12. Both the siRNA-ONV and 2nd-siRNA-ONV demonstrate similar gene silencing, protein expression and cell proliferation inhibition effects under serum condition compared with serum-free condition, suggesting serum did not affect the “double locks-smart key” controlled siRNA delivery. The detailed information on the experiment has been added in page 22 lines 5-8, and discussed in page 12 lines 16-21.

**Reviewer #3 (Remarks to the Author):**

The authors addressed my concerns to my general satisfaction. What were the difficulties in detecting correctly processed siRNAs by the suggested detection kits? One limitation to consider is that the detection of a band in a cell lysate does not necessarily prove correct RNA processing in an intact cell.

The Methods section does not provide the sequence of the second active VEGF siRNA. Instead, it repeats the sequence of the first one. I am assuming that this is an error and that the sequence of the second siRNA is clearly different from the first one due to targeting a distinct site in the VEGF transcript. The authors must correct this error prior to publication.

**To Reviewer 3:**

1). *The authors addressed my concerns to my general satisfaction. What were the difficulties in detecting correctly processed siRNAs by the suggested detection kits? One limitation to consider is that the detection of a band in a cell lysate does not necessarily prove correct RNA processing in an intact cell.*

**Response:** Thank you very much for bringing the concern. Based on our knowledge, the commercial siRNA detection kit adds polydA to d(TT) 3' overhang of siRNA and carries out a reverse transcription reaction with a specific oligo dT primer, in combination with realtime PCR analysis for quantification. In this manuscript, the d(TT) 3' overhang of VEGF siRNA was always exposed and could initiated the above reaction no matter it conjugated with delivery vehicle ONV or released as free siRNA in cytoplasm. Therefore, the detection kit could only detect the total amount of siRNA, and hardly characterize its release process.

The cell lysate derived from the intact cell, which should also reflect the ability of intact cell. The *in vitro* PAGE assays of the mixture of oligodeoxynucleotide and cell lysate have been used to demonstrate the intended cleavage of target RNA in the intact cell (*Biochim. Biophys. Acta* **2009**, *1790*, 1170–1178; *Eur. J. Biochem.* **2002**, *269*, 583-592). Therefore, we also used the *in vitro* PAGE assay to demonstrate the release of siRNA in cytoplasm. The above relevant references have been added as refs 42 and 43 in page 27 lines 4-8.

2). *The Methods section does not provide the sequence of the second active VEGF siRNA. Instead, it repeats the sequence of the first one. I am assuming that this is an error and that the sequence of the second siRNA is clearly different from the first one due to targeting a distinct site in the VEGF transcript. The authors must correct this error prior to publication.*

**Response:** Thank you very much for your kind and valuable reminding. The sequences for the second siRNA is: sense 5'-ACCUCACCAAGGCCAGCACdTdT-3', antisense 5'-GUGCUGG CCUUGGUGAGGUdTdTCAAAUGGACCAAGGCCAG-3', and has been corrected in page 16 lines 9-11.
